# Supplementary material for: Language models and protocol standardization guidelines for accelerating synthesis planning in heterogeneous catalysis
Source: Nat Commun. 2023 Dec 2;14:7964. doi: 10.1038/s41467-023-43836-5 (PMC10693572; doi:10.1038/s41467-023-43836-5)
Supplement: Supplementary file 1 — Supplementary Information [file 41467_2023_43836_MOESM1_ESM.pdf]

Supplementary Information

Language models and protocol standardization guidelines for accelerating synthesis planning in heterogeneous catalysis

Manu Suvarna[1], Alain Claude Vaucher[2], Sharon Mitchell[1], Teodoro Laino[2,*], and Javier Pérez-Ramírez[1,*]

[1]Institute for Chemical and Bioengineering, Department of Chemistry and Applied Biosciences, ETH Zurich, Vladimir-Prelog-Weg 1, 8093 Zurich, Switzerland.

[2]IBM Research Europe, Säumerstrasse 4, 8803 Rüschlikon, Switzerland.

*Corresponding authors. E-mails: jpr@chem.ethz.ch; teo@zurich.ibm.com

Table of contents

- Supplementary Note 1. Classification of single-atom catalysts synthesis procedures 1
- Supplementary Table 1. Classification of single-atom catalysts synthetic methods 2
- Supplementary Fig. 1. Treemaps representing the various synthesis methods 3
- Supplementary Note 2. Defining action terms for synthesis procedures 4
- Supplementary Table 2. Action terms in SAC synthesis protocols and description 5
- Supplementary Table 3. Relevant information or parameters and corresponding data types associated with each action term 6
- Supplementary Table 4. Examples of the annotation process 8
- Supplementary Note 3. Model architecture and implementation 9
- Supplementary Table 5. Hyperparameters of Organic model 10
- Supplementary Table 6. Hyperparameters of ACE model 11
- Supplementary Note 4. Predictive performance of the ACE model 12
- Supplementary Fig. 2. Performance analysis of the ACE model 13
- Supplementary Note 5. Comparative analysis of the ACE and pretrained models 14
- Supplementary Table 7. Comparison of the ACE and pretrained models for SAC synthesis protocol extraction 15
- Supplementary Note 6. Model generalization 16
- Supplementary Note 7. Model limitations 38
- Supplementary Note 8. Model predictions with and without protocol standardization 42

Supplementary Note 1. Classification of single-atom catalyst synthesis procedures

In efforts to synthesize stable single atoms in selected architectures, researchers have explored and reported various synthetic routes, including wet-chemical, solid-state, and gas-phase routes among others.[1–3] At the very beginning of the study, it was essential to gain preliminary insights on the trends in SAC synthetic routes as these encompass various steps, such as mixing, wet deposition, pyrolysis, filtering, washing, annealing, etc*.* An understanding of the most commonly used synthetic routes, would facilitate identification of action terms that encode relevant information of synthesis steps, and are essential for training of language-based models. For this purpose, we manually analyzed 145 articles across thermo-, electro-, and photocatalytic reactions and categorized the synthesis procedures explored in these publications into 8 broad categories and an additional post-synthetic treatment step (**Supplementary Table 1**). The subset of 145 paragraphs were selected without any bias or *a-priori* knowledge. Based on the outcome of our analysis, we observe that solution-phase and high-temperature synthesis or their hybrid forms were the most representative accounting for close to 80% of the total (**Supplementary Fig. 1**).

Supplementary Table 1. Classification of single-atom catalysts synthetic methods.

Synthetic methods	Approaches covered	Description
High-temperature synthesis	• Pyrolysis<br>• Thermal decomposition<br>• Carbonization<br>• Polymerization	Synthesis of SAC by thermal treatment (T>573 K)
Gas and vapor phase synthesis	• Chemical vapor deposition<br>• Physical vapor deposition<br>• Molecular beam epitaxy<br>• Pulsed laser deposition	Synthesis of SAC in the gas phase or by deposition of a vaporized precursor
Solution-phase synthesis	• (Wet/dry) Impregnation<br>• Wet deposition<br>• Ion exchange<br>• Precipitation<br>• Hydrothermal<br>• Colloidal synthesis	Synthesis of SAC in solution phase
Electrochemical synthesis	• Electrodeposition<br>• Galvanic displacement	Synthesis of SAC through electrochemical reactions
Photochemical synthesis	• Photoreduction<br>• Photodeposition<br>• Photocatalytic	Synthesis of SAC using light as a source of energy
Mechanochemical synthesis	• Grinding<br>• Milling<br>• Shearing	Synthesis of SAC using mechanical energy
Templated synthesis	• Molecular templates<br>• Porous material templates	Uses templates to guide the atomic dispersion of the metal or tailor the properties, such as porosity, of the support
Hybrid synthesis	• For example, high-temperature and solution phase	Combines more than one synthetic approach
Post-synthetic treatments	• Oxidation<br>• Reduction<br>• Heating/Drying<br>• Annealing<br>• Acid leaching<br>• Support modification<br>• Addition of promoter	Refer to processes applied after synthesis to tune the SAC properties or remove unwanted species (metal clusters or byproducts). While they are separate steps from synthesis, they are often interdependent

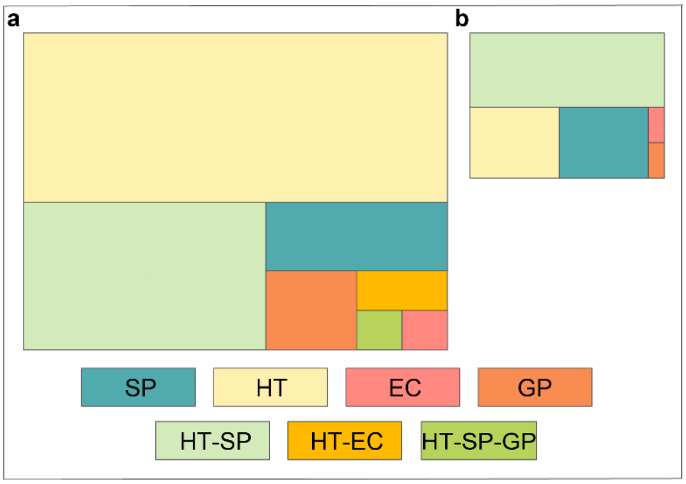

Supplementary Fig. 1. Treemaps representing the various synthesis methods investigated for **a)** electrocatalytic and **b)** thermocatalytic reactions. The area of the treemaps are scaled in percentages to the respective reaction classes. Abbreviations included are SP: solution-phase, HT, high-temperature; EC, electrochemical; GP, gas phase; and their corresponding hybrid methods.

Supplementary Note 2. Defining action terms of synthesis procedures

When reading articles, it is easy to notice that researchers use diverse language and give different levels of details when reporting heterogeneous catalyst synthesis protocols. This lack of standardization, which can make it challenging to compare data and reproduce experiments, most likely stems from the limited guidelines available. For example, journals typically specify that experimental descriptions should enable others to be able to replicate and build upon the authors' published claims. However, this statement leaves it open to interpretation what synthetic knowledge other researchers have. Furthermore, word limits may force researchers to only provide minimal details, while self-plagiarism considerations prompt researchers to be creative with how they report standardized procedures to avoid using the same wording. These factors result in a colorful variety of language used to describe similar synthetic approaches and a wide variety of action terms, which we are interested in extracting from the synthesis protocols through text mining.

In this study, we used 33 action terms (**Supplementary Table 2**) used to describe the synthesis of heterogeneous catalysts and important information or criteria associated with each (**Supplementary Table 3**). In deciding how many action terms to define, we aimed to provide a comprehensive set of the common synthetic steps, for instance solution-phase and high-temperature procedures, while ensuring a practical size of the model to facilitate its training. To assist readers with the application of these action types, we have provided examples in **Supplementary Table 4**.

Supplementary Table 2. Action terms in SAC synthesis protocols and their description.

#	Action term[a,b]	Description
1.	Add	Add a substance to the synthesis vessel
2.	Centrifuge*	Centrifuge the synthesis mixture
3.	CollectLayer	Isolate the aqueous or organic fraction
4.	Concentrate	Evaporate the solvent
5.	Degas	Purge with a gas
6.	DrySolid	Dry a solid
7.	DrySolution	Dry an organic solution
8.	Extract	Transfer compound into a different solvent
9.	Filter	Phase to keep either after filtration or centrifugation
10.	FollowOtherProcedure	The text refers to a procedure described elsewhere
11.	Grind*	Grind a solid material/mixture
12.	InvalidAction	Unknown or unsupported action
13.	Leach*	Detach from the carrier
14.	MakeSolution	Mix two or more substances to create a solution or dispersion
15.	Microwave	Heat using microwave irradiation
16.	NoAction	The text contains no action
17.	OtherLanguage	The text is not written in English
18.	Partition	Add two immiscible solvents for phase separation
19.	pH	Adjust or maintain the pressure of the reaction mixture
20.	PhaseSeparation	Separate the aqueous and organic phases
21.	Purify	Purification via chromatography
22.	Quench	Stop reaction by adding a substance
23.	Recrystallize	Recrystallize a solid from a solvent or mixture of solvents
24.	Reflux	Reflux the reaction mixture
25.	SetTemperature	Adjust or maintain the temperature of the reaction mixture
26	Sonicate	Agitate the solution with sound waves
27.	Stir	Stir for a specified duration
28.	SynthesisProduct*	Indicates the chemical identity/name of the catalyst
29.	ThermalTreatment*	A treatment applied during or post synthesis such as heating, carbonization, pyrolysis, calcination, oxidation, and annealing
30.	Transfer*	Transfer a compound to designated reaction vessel
31.	Wait	Leave to stand for a specified duration
32.	Wash	Wash liquid or solid with a solvent
33.	Yield	The product of an intermediate step or final catalyst

[a] The initial set of 27 action terms are reported elsewhere.[4] [b] The action terms introduced to extend the model in this study are marked with an asterisk.

Supplementary Table 3. Relevant information or parameters and corresponding data types associated with each action term.

Action term	Information/Parameter	Data type [a]
Add	Material	Chemical
	Dropwise	Boolean
	Temperature	String (optional)
	Atmosphere	String (optional)
	Duration	String (optional)
Centrifuge	Duration	String (optional)
	RPM	String (optional)
	Temperature	String (optional)
CollectLayer	Layer	String
Concentrate	None	
Degas	Gas	String
DrySolid	Duration	String (optional)
	Temperature	String (optional)
	Atmosphere	String (optional)
DrySolution	Material	String
Extract	Solvent	Chemical
	Repetitions	Integer
Filter	Phase_to_keep	String (optional)
FollowOtherProcedure	None	
Grind	Duration	String (optional)
	Material	String (optional)
	Temperature	String (optional)
InvalidAction	Error	String
Leach	Duration	String (optional)
	Material	String (optional)
	Material_quantitiy	String (optional)
	Temperature	String (optional)
MakeSolution	Material_1	String
	Material_2	String
	Material_3	String
	Material_4	String
	Material_5	String
	Material_6	String
	Material_7	String
	Material_8	String
	Material_9	String
	Material_10	String
	Material_quantity_1	String (optional)
	Material_quantity_2	String (optional)
	Material_quantity_3	String (optional)
	Material_quantity_4	String (optional)

	Material_quantity_5	String (optional)
	Material_quantity_6	String (optional)
	Material_quantity_7	String (optional)
	Material_quantity_8	String (optional)
	Material_quantity_9	String (optional)
	Material_quantity_10	String (optional)
Microwave	Duration	String (optional)
	Temperature	String (optional)
NoAction	None	
OtherLanguage	None	
Partition	Material_1	Chemical
	Material_2	Chemical
pH	Material	Chemical
	pH	String (optional)
	Dropwise	Boolean
PhaseSeparation	None	
Purify	None	
Quench	Material	Chemical
	Dropwise	Boolean
	Temperature	String (optional)
Recrystallize	Solvent	Chemical
Reflux	Duration	String (optional)
	atmosphere	String (optional)
SetTemperature	Temperature	String
Sonicate	Duration	String (optional)
	Temperature	String (optional)
Stir	Duration	String (optional)
	Temperature	String (optional)
	atmosphere	String (optional)
SynthesisProduct	Material	Chemical
ThermalTreatment	Type	String (optional)
	Atmosphere	String (optional)
	Temperature	String (optional)
	Ramp	String (optional)
	Duration	String (optional)
Transfer	Quantity	String (optional)
	Vessel	String (optional)
Wait	Duration	String
	Temperature	String (optional)
Wash	Material	Chemical
	Repetitions	Integer
Yield	Material	Chemical

[a] The data type “Chemical” comprises a string for the chemical name and a (possible empty) list of quantities (as strings).

Supplementary Table 4. Examples of the annotation process of commonly occurring sentences in synthesis paragraphs.

Sentence	Annotation example
2.32 mmol D-glucosamine hydrochloride and 0.464 mmol ammonium iron(II) sulfate hexahydrate were co-dissolved in 25 mL Te nanowire solution.	Annotated as MakeSolution<br>This action has two inherent parameters i) material and ii) material_quantity<br>Details extracted: Material - D-glucosamine hydrochloride, ammonium iron(II) sulfate hexahydrate or Te nanowire solution, material_quantity - 2.32 mmol, 0.464 mmol and 25 mL
The pyrolysis of the dried fluffy powder was performed at 900°C for 1 h under argon atmosphere with heating rate of 2°C min^{-1}.	Annotated as ThermalTreatment<br>This action has five parameters i) type, ii) atmosphere, iii) temperature, iv) ramp, and v) duration<br>Details extracted: Type - pyrolysis, atmosphere - argon, temperature - 900°C, ramp - 2°C min^{-1} and duration - 1 h
The reaction mixture was cooled to room temperature over 5 h.	Annotated as SetTemperature<br>This action has two relevant parameters (i) temperature and (ii) duration<br>Details extracted: temperature - room temperature, duration - 5 h
The synthesized catalyst was denoted as 0.04 wt% Au_1/CeO_2.	Annotated as SynthesisProduct or Yield<br>The targeted catalyst is typically listed at the beginning or end of the synthesis paragraph<br>Details extracted: Au_1/CeO_2
The actual Pt loadings of the samples were determined by inductively coupled plasma spectroscopy on an IRIS Intrepid II XSP instrument.	Annotated as NoAction<br>Sentences does not describe a synthetic step<br>Details extracted: None
All the reagents in this study were of analytical grade and used without further purification unless specified.	Annotated as NoAction<br>Sentences does not describe a synthetic step<br>Details extracted: None

Supplementary Note 3. Model architecture and implementation

The ACE model is implemented using the OpenNMT-py library, version 1.1.1.[5] The architecture has eight attention heads, and the model is trained by minimizing the categorical cross-entropy loss for the output subwords. The model was trained for 500,000 steps, with selected hyperparameters (**Supplementary Table 5**), while the parameters used for fine-tuning the ACE model are shown in (**Supplementary Table 6**). Several experiments were run to optimize the hyperparameters including learning_rate and warmup_steps. The models were trained for 20,000 steps, with the checkpoint at 9,000 steps identified as the best-performing model on the validation set.

The paragraph-to-sentence segmentation, which is the very core of our model, is handled modularly by making use of Python class called 'SentenceSplitter' and can be found in our repository: https://github.com/rxn4chemistry/paragraph2actions/blob/main/src/paragraph2actions/sentence_splitting/sentence_splitter.py. This allows for segmenting the synthesis procedures into single sentences reliably. However, we highlight that some cross-dependencies over multiple sentences do occur and information about a specific action item could spread over two sentences. In such scenario's, based on our previous observations,[3] we note that information contained in consecutive sentences could also be determined from context when analyzing the action terms.

Once the model translates the paragraphs into a sequence of actions, the results are converted to Python objects with all the associated properties as listed in **Supplementary Table 3**. We have developed user-defined functions for extracting various parameters such as compound names, quantities, or temperatures etc., the details of which can be found in out GitHub repository: https://github.com/rxn4chemistry/sac-action-extraction#analysis-of-extracted-actions

Supplementary Table 5. Hyperparameters that were used for pretraining the model Parameters not affecting the model architecture or the training process are not listed.

Parameter	Value
accum_count	4
adam_beta1	0.9
adam_beta2	0.998
batch_size	4096
batch_type	tokens
decay_method	noam
decoder_type	transformer
dropout	0.1
encoder_type	transformer
global_attention	general
global_attention_function	softmax
heads	8
label_smoothing	0
layers	4
learning_rate	2
max_generator_batches	32
max_grad_norm	0
normalization	tokens
optim	adam
param_init	0
Param_init_glorot	True
Position_encoding	True
rnn_size	256
seed	42
self_attn_type	scaled-dot
share_embeddings	True
transformer_ff	2048
warmup_steps	8000
word_vec_size	256

Supplementary Table 6. Hyperparameters that were used for fine-tuning the ACE model. Parameters that do not affect the model architecture or the training process are not listed.

Parameter	Value
accum_count	4
adam_beta1	0.9
adam_beta2	0.998
batch_size	4096
batch_type	tokens
decay_method	noam
dropout	0.1
label_smoothing	0
learning_rate	0.2
max_generator_batches	32
max_grad_norm	0
normalization	tokens
optim	adam
reset_optim	all
seed	42
warmup_steps	8000

Supplementary Note 4. Predictive performance of the ACE model

We assessed the model performance by devising two scenarios *i.e. i*) effect of dataset size *ii*) data augmentation and benchmarked them with respect to the pre-trained model by using metrics, such as the BLEU (BiLingual Evaluation Understudy) and Levenshtein similarity score. In the first scenario, we evaluate the performance by progressively including 25%, 50%, 75%, and 100% of the training split from the annotated data. Here, we observe a drastic increase in performance even when including only 25% of the annotated data compared to the pre-trained model. However, subsequent increase in the size of annotations in the training sample from 25% to 100% resulted in a mere (ca. 2%) increase in the Levenshtein score from 62% to 63.8%, respectively. We also assessed the effect of data augmentation, where the dataset size was synthetically increased by a factor 10 by substituting precursor and solvent names along with their quantities, unit operations, and thermal treatment details. Under this scenario, and the model performance improved to 65% and 66% when using 50% and 100% annotations, respectively.

For each of the 8 training runs performed across both the scenarios, the metrics on the test set and the corresponding checkpoint leading to the highest accuracy in the validation set are shown in (**Supplementary Fig. 2a,b**). As a general trend, we observe that metrics improve with additional data in both the scenarios, and data augmentation positively impacts the model's performance, which is consistent with literature reports.[4,6] The best Levenshtein similarity of 0.66 represents the normalized score of the model, implying that the model can capture and extract approximately 66% of information on the test sets, while a BLEU score of above 50 implies high quality translation of synthesis sentences in natural language to machine readable formats. Despite training on a much larger dataset in the original work,[4] the pre-trained model consistently showed worse performance metrics than the ACE counterpart. This implies that predicting the entire sequence of synthesis actions is non-trivial and necessitates domain-specific annotation.

a

Model	Annotation %	Data augment	BLEU score	LSH score
ACE	25	-	47.9	62.0
ACE	50	-	52.1	63.0
ACE	75	-	49.9	64.0
ACE	100	-	51.9	63.8
ACE	25	10-fold	50.3	62.7
ACE	50	10-fold	51.4	65.1
ACE	75	10-fold	51.8	65.2
ACE	100	10-fold	52.0	66.1
Pretrained	0	-	19.6	41.5

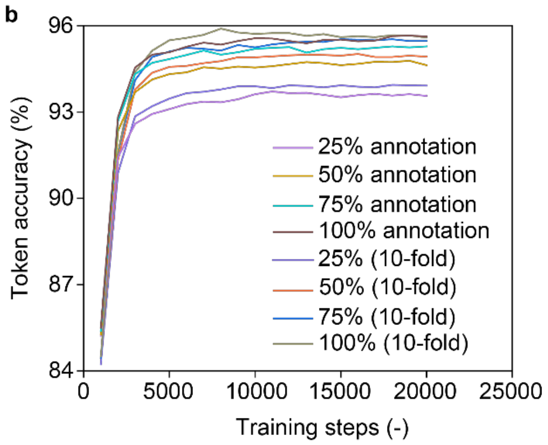

Supplementary Fig. 2. Performance analysis of the ACE model. a) Comparative evaluation of the ACE on SAC protocols on the test set under varying dataset size and data augmentation strategies. All scores are given in %. Abbreviations used include BLEU, BiLingual Evaluation Understudy; and LSH, Levenshtein. **b)** Training curves for the ACE model under eight scenarios. The Y-axis and X-axis represent the average number of tokens predicted correctly and the required number of training steps, respectively when evaluated on the validation set.

Supplementary Note 5. Comparative analysis of the ACE and Organic models

In the previous publication, the Organic model was developed for extracting synthesis protocols of organic reactions by transfer-learning on a pretrained model. The ACE model was obtained in a similar way from the same pretrained model, by considering additional annotations related to the synthesis protocols of heterogeneous catalysts. The main difference between these models is that the latter is trained on specific action terms (**Supplementary Table 2**) while the former is not, leading to differences in their prediction capabilities.

To illustrate the differences in performance, we considered the following example paragraph: In a typical procedure, nickel nitrate hexahydrate (3.48 g) and sodium citrate (4.71 g) were dissolved in 400 mL water to form clear solution A. In the meantime, potassium hexacyanocobaltate (III) (2.66 g) was dissolved into water (200 mL) to form clear solution B. Then, solutions A and B were mixed under magnetic stirring. Stirring was stopped after combining the component solutions. After 24 h, the solid was collected by centrifugation, washed with water and ethanol, and then dried at room temperature. Then, the dried sample was annealed at 500°C in NH_3 for 1 h with a slow heating rate of 2°C min^{-1}, and immersed in 5 M H_2SO_4 solution and stirred at 80°C for 24 h to form CoNi-SAs/NC hollow cubes.[7]

We analyzed the output of the ACE and pretrained models for this paragraph in **Supplementary Table 7**. While both models extract relevant information in similar sequential steps, the pretrained model made four incorrect extraction steps. The first three reflect its lack of knowledge of specific action terms such as ThermalTreatment, which were added when developing the ACE model for heterogeneous catalyst synthesis. Additionally, the pretrained model incorrectly predicted the targeted catalyst, mistaking Co for the number 4. In contrast, the ACE model extracted the synthetic protocol more precisely, making it easier for experimental researchers or machines to interpret the data extracted.

Supplementary Table 7. Comparison of the ACE and Organic models for SAC synthesis protocol extraction.

ACE model[a]	Organic model[a]
1. MakeSolution with nickel nitrate hexahydrate (3.48 g) and sodium citrate (4.71 g) and water (400 mL)	1. Add nickel nitrate hexahydrate (3.48 g)
2. Yield solution A	2. Add sodium citrate (4.71 g)
3. MakeSolution with potassium hexacyanocobaltate (III) (2.66 g) and water (200 mL)	3. Add water (400 mL)
4. Yield solution B	4. Add potassium hexacyanocobaltate (III) (2.66 g)
5. Add solutions A	5. Add water (200 mL)
6. Add B	6. Add solutions A
7. Stir	7. Add B
8. Wait for 24 h	8. **CollectLayer organic**
9. Centrifugate	9. Wait for 24 h
10. Wash with water	10. Filter keep precipitate
11. Wash with ethanol	11. Wash with water
12. DrySolid at room temperature	12. Wash with ethanol
13. ThermalTreatment annealing under NH_3 for 1 h at 500°C ramp 2°C min^{-1}	13. DrySolid at room temperature
14. Add H_2SO_4 (5 M)	14. **Stir for 1 h at 500°C**
15. Stir for 24 h at 80°C	15. **Add NH_3**
16. Yield CoNi-SAs/NC	16. Stir for 24 h at 80°C
	17. **Yield 4-Ni-SAs/NC**

[a] Incorrect predictions are indicated in **bold** text.

Supplementary Note 6. Model generalization

In this section, we show that the predictive and extraction capabilities of the ACE model are not limited to SAC synthesis procedures alone but extend to other classes of heterogeneous catalysts. The model's data-driven nature and training on action terms covering a broad spectrum of relevant synthetic operations allow it to scale its predictive capabilities to various classes of catalytic materials. To demonstrate this, we selected 18 synthesis paragraphs covering catalysts for different applications including CO_2 hydrogenation to methanol, Fischer-Tropsch synthesis, and acetylene hydrochlorination. The synthesis paragraphs for these reactions are presented in prose, and the model output in presented as a sequence of 202 action items listed in bullet points. It is noteworthy that of the total action items, model is able to predict 125 actions correctly inclusive of all essential details and 28 actions partially correct. As such, it can capture most of the relevant synthetic steps resulting in a human machine-readability index of 69%. The few incorrect predictions *i.e.* 49 actions are consistent throughout (including SAC paragraphs) and can be attributed due to lack of specific action terms, and in most cases the incorrect terms are less relevant. For the examples provided below, the catalyst synthesis recipes are directly copied from the referenced articles, the output of the model is bulletized, and incorrect model predictions are highlighted in **bold**.

Cl promoted Fe catalyst for Fischer-Tropsch synthesis

Typically, a mixture of an $Fe(NO_3)_3 \cdot 9H_2O$ and $Cu(NO_3)_2 \cdot 3H_2O$ aqueous solution ($c(Fe^{3+}) = 0.4\ mol \cdot L^{-1}$, $c(Cu^{2+}) = 3.5 \times 10^{-3}\ mol \cdot L^{-1}$) and an aqueous Na_2CO_3 solution ($c(Na^+) = 2.4 \times 10^{-3}\ mol \cdot L^{-1}$) were added together into a beaker by two pumps at a constant flow rate. The obtained suspension was centrifuged and washed with deionized water three times. The filter cake obtained was then pulped with a K_2SiO_3 solution ($c(K^+) = 8\ mol \cdot L^{-1}$, $m(Fe):m(K_2SiO_3) = 1:1$). The mixture was then spray-dried and calcined at 600°C for 6 h to obtain the precipitated iron Fischer–Tropsch synthesis catalyst. The precipitated iron Fischer–Tropsch synthesis catalysts modified with chlorine were prepared by the impregnation method. The precipitated iron Fischer–Tropsch synthesis catalysts were impregnated with an aqueous $FeCl_3 \cdot 6H_2O$ solution with different concentrations and dried at 120°C overnight. Depending on the quantity of the chlorine addition, the obtained catalysts were denoted as Cl01, Cl1, and Cl5, where the numbers represented the percentage of chlorine impregnated. As a comparison, a catalyst impregnated by deionized water was also prepared, which was represented as Cl0.[8]

- MakeSolution with $Fe(NO_3)_3 \cdot 9H_2O$ and $Cu(NO_3)_2 \cdot 3H_2O$ aqueous solution **(0.4 mol·L^{-1})** and aqueous Na_2CO_3 solution (c(Na^+ = 2.4 mol·L^{-1})
- Centrifuge
- Wash with water 3 x
- **Wash with K_2SiO_3 solution (c(K^+) (8 mol·L^{-1})**
- DrySolid under freeze spray-dried
- ThermalTreatment calcination under None for 6 h at 600°C
- Yield iron **Fer-Tropsch catalyst**
- FollowOtherProcedure
- **Wash with aqueous $FeCl_3 \cdot 6H_2O$ solution**
- DrySolid for overnight at 120°C
- **Add chlorine**

- **Add CI1**
- **Add CI5**
- FollowOtherProcedure

K-Fe/NC catalyst for Fischer-Tropsch synthesis

PB as precursors for the subsequent experiments was prepared by a simple precipitation method, as reported in the literature. Firstly, solution A was obtained by adding 0.6 mmol of $FeSO_4 \cdot 6H_2O$ and 0.9 mmol of $Na_3C_6H_5O_7 \cdot 2H_2O$ into 20 ml of deionized water. Secondly, solution B was obtained by adding 0.4 mmol of $K_3Fe(CN)_6$ into 20 ml of deionized water. Thirdly, solution B was added slowly into solution A under vigorous and continuously stirring, and then the obtained mixture was aged at room temperature for 24 h. Finally, the aged mixture was treated by centrifugation, washing with water and ethanol, and drying at 60°C overnight. The PB powder was obtained. The as-prepared PB powder was further pyrolyzed at 500°C for 4 h with a heating rate of 3°C min^{-1} under a flow of N_2 atmosphere. After the pyrolysis process, the temperature was decreased to room temperature under N_2 atmosphere, and then the N_2 was changed to air for the passivation of the K-Fe/NC catalyst. Finally, the K - Fe/NC-fresh-P catalyst was obtained.[9]

- FollowOtherProcedure
- MakeSolution with $FeSO_4 \cdot 6H_2O$ (0.6 mmol) and $Na_3C_6H_5O_7 \cdot 2H_2O$ (0.9 mmol) and deionized water (20 ml)
- MakeSolution with $K_3Fe(CN)_6$ (0.4 mmol) and deionized water (20 ml)
- Add solution A
- Stir for 24 h at room temperature
- Centrifuge
- Wash with water
- Wash with ethanol
- DrySolid for overnight at 60°C
- ThermalTreatment pyrolysis under N_2 for 4 h at 500°C ramp 3°C min^{-1}
- NoAction
- Yield K-Fe/NC-fresh-P catalyst

Co/SiO_2 catalysts for Fischer-Tropsch synthesis

The Co/SiO_2 catalyst containing 20 wt% Co was prepared by incipient wetness impregnation. Briefly, 10 g SiO_2 (200 m^2g^{-1}, Evonik) was impregnated with an aqueous solution of $Co(NO_3)_2.6\ H_2O$. The resulting sample was then dried at 100°C for 12 h, followed by calcination in air at 350°C for 5 h. The calcined catalyst was reduced at 400°C for 5 h using 10 % H_2/N_2 (200 mL min^{-1}). The as-obtained sample was denoted as R. The R sample was further carburized by 97 % CO/N_2 under 220°C with gas flow rate of 50 mL min^{-1} for 5 h, 10 h and 24 h to obtain the RC-5, RC-10 and RC-24 samples, respectively.[10]

- **NoAction**
- **NoAction**
- DrySolid for 12 h at 100°C
- ThermalTreatment calcination under air for 5 h at 350°C
- ThermalTreatment calcination under None for 5 h at 400°C
- **SynthesisProduct 10 % H_2/N_2 (200 mL min^{-1})**
- ThermalTreatment heat under hydrogen for 5 h at 220°C ramp 50 mL min^{-1}
- Wait for 24 h
- Yield RC-10 and RC-24

Fe/SiO_2 catalysts for Fischer-Tropsch synthesis

Fe/SiO_2 catalysts were prepared by a wet impregnation method using $Fe(NO_3)_3 \cdot 9H_2O$ (Sigma-Aldrich, ≥98%) and SiO_2 (Sigma-Aldrich, high-purity grade, S_{BET} = 450 $m^2\ g^{-1}$, average pore diameter = 4 nm). $Fe(NO_3)_3 \cdot 9H_2O$ was dissolved in deionized (D.I.) water and mixed with SiO_2. The mass ratio of aqueous $Fe(NO_3)_3 \cdot 9H_2O$ to SiO_2 was 10. The mixture was stirred at 60°C for 6 h at 120 rpm and evaporated using a rotary evaporator (IKA, RV 10 Digital V). The final product was dried overnight at 110°C and calcined at 550°C for 4 h at a heating rate of 4°C min^{-1}. For the preparation of $Fe\text{-}SiO_2$, the Fe/SiO_2 catalyst was melt-fused in an alumina crucible at 1700°C (T_m of Fe_2O_3 = 1550°C, T_m of SiO_2 = 1670°C) for 6 h in air at a heating rate of 10°C min^{-1}.[11]

- **NoAction**
- Add $Fe(NO_3)_3 \cdot 9H_2O$
- Add (D.I.) water
- Add SiO_2
- **NoAction**
- Stir for 6 h at 60°C
- Concentrate
- DrySolid for overnight at 110°C
- ThermalTreatment calcination under None for 4 h at 550°C ramp 4°C min^{-1}
- **InvalidAction**

Supported CoMn catalyst for Fischer-Tropsch synthesis

The CoMn catalyst with a Co/Mn (mol/mol) atomic ratio of 2/1 was prepared with a coprecipitation method. $Co(NO_3)_2 \cdot 6H_2O$, 50 wt% $Mn(NO_3)_2$ and $(NH_4)_2CO_3$ solution (analytical grade) were purchased from Sinopharm Chemical Reagent Co., Ltd. and then dissolved in deionized water to form a 2 M mixed salt solution. The solution containing 2 M total metal ions and saturated $(NH_4)_2CO_3$ was added simultaneously into the mother solution (100 mL deionized water) as a precipitant under strong stirring in a beaker. The pH value of the solution was fixed at about 8, and the temperature of the solution was kept at about 30°C. After titration, the as-prepared mixture was aged at 30°C for 2 h under continuous stirring, then washed several times with deionized water until the pH value of the centrifugal liquid reached *ca.* 7.0, followed by being dried at 100°C for 10 h and then calcined at 330°C for 3 h in static air.[12]

- **FollowOtherProcedure**
- **NoAction**
- **Add 2 Mansfer to saturated $(NH_4)_2CO_3$ (2 M)**
- Stir
- SetTemperatureabout 30°C
- Stir for 2 h at 30°C
- Wash with deionized water 5 x
- DrySolid for 10 h at 100°C
- ThermalTreatment calcination under air for 3 h at 330°C

Zr-promoted Fe catalyst for Fischer-Tropsch synthesis

FeMn*x*Zr catalysts were prepared by coprecipitation methods. Briefly, a solution contains a desired ratio of $Fe(NO_3)_3 \cdot 9H_2O$ (99.99 wt% purity, Macklin), $Mn(NO_3)_2$ (50 wt% purity, Macklin), and $Zr(NO_3)_4 \cdot 5H_2O$ (99% wt% purity, Macklin) which were used as metal sources. Ammonia solution (25- 28%, AR, Aladdin) was used as the precipitant. The mixed metal nitrate solution and ammonia solution were added dropwise into a beaker at the same time at a temperature of 65°C, stirring continuously. The pH of the mixed solution was controlled at 8.0 ± 0.1 and the stirring was continued for 1 h. The precursors were aged for 4 h, washed with deionized water, dried at 110°C for 12 h, and then calcined at 500°C for 4 h. The coprecipitated FeMn*x*Zr catalysts with molar compositions of 100Fe/5Mn/*x*Zr (*x* = 0, 1, 3, 5, and 10) were labeled FeMn, FeMn1Zr, FeMn3Zr, FeMn5Zr, and FeMn10Zr, respectively.[13]

- **FollowOtherProcedure**
- NoAction
- Add metal nitrate solution at 65°C
- Add ammonia solution at 65°C
- Stir for 1 h at pH 8.0 ± 0.1
- Wait for 4 h
- Wash with deionized water
- DrySolid for 12 h at 110°C
- ThermalTreatment calcination under None for 4 h at 500°C
- **NoAction**

Promoted Fe catalyst for Fischer-Tropsch synthesis

The transition metals promoted iron-based catalysts were fabricated by a coprecipitation method. In a typical preparation, 500 mL NaOH solution (1.5 mol L^{-1}) was added dropwise into 150 mL aqueous solution containing 0.12 mol of $Fe(NO_3)_3 \cdot 9\ H_2O$ and 0.04 mol of $M(NO_3)_x$ (M = Zn, Cu or Mn) under vigorous stirring at 60°C until the pH value reached around 9. Afterwards, the obtained slurry was aged for 1 h, and washed with deionized water to control the residual content of Na. Subsequently, the filtered cakes were dried at 80°C overnight, and calcined at 400°C for 3 h. The as-prepared catalysts were denoted as FeZn-Na, FeCu-Na and FeMn-Na. For comparison, Fe-Na catalysts was also synthesized by the same procedure but without the addition of $M(NO_3)_x$.[14]

- **NoAction**
- **InvalidAction**
- MakeSolution with $Fe(NO_3)_3 \cdot 9\ H_2O$ (0.12 mol) and $M(NO_3)x$ (0.04 mol) and **Cu or Mn**
- Add solution dropwise at 60°C
- Wait for 1 h
- Wash with deionized water
- DrySolid for overnight at 80°C
- ThermalTreatment calcination under None for 3 h at 400°C
- SynthesisProduct FeZn-Na
- FollowOtherProcedure

Transition metal carbides for CO_2 hydrogenation

First, 17.92 g of MeIm was dissolved in 280 mL of deionized water. Next, 580 mg of $Co(NO_3)_2·6H_2O$ and 30 mg of CTAB were dissolved in another 20 mL of deionized water. Then, the mixture of $Co(NO_3)_2·6H_2O$ and CTAB was added into the MeIm solution and was stirred for 20 min. The bright purple product was collected by centrifugation, washed several times with ethanol, and dried at 80°C for 12 h. The resulting ZIF-67 sample was heated to 350°C for 2 h at a heating rate of 0.5°C min^{-1} in a muffle furnace. The sample was prepared by the incipient wetness impregnation method. Co_3O_4-HC (0.5 g) was impregnated with an aqueous solution of alkali metals, in which the loading was maintained at 1 wt%, and then dried at 60°C for 12 h.[15]

- MakeSolution with MeIm (17.92 g) and deionized water (280 mL)
- MakeSolution with $Co(NO_3)_2·6H_2O$ (580 mg) and CTAB (30 mg) and deionized water (20 mL)
- MakeSolution with $Co(NO_3)_2·6H_2O$ and CTAB
- Add SLN
- Stir for 20 min
- Centrifuge
- Wash with ethanol 5 x
- DrySolid for 12 h at 80°C
- ThermalTreatment heat under None for 2 h at 350°C ramp 0.5°C min^{-1}
- **NoAction**
- **Add** Co_3O_4-HC (0.5 g)
- **Add** aqueous solution of alkali metals
- DrySolid for 12 h at 60°C

Supported Cu-based catalyst for methanol synthesis

A series of $CuZnO_x/C$ catalysts, with similar Cu weight loadings (8.0 ± 0.4 wt%) but varying Zn/Cu molar ratios, were prepared via incipient wetness impregnation following a published method. In brief, powdered high-surface-area graphite (TIMREX E-HSAG500, TIMCAL Graphite & Carbon) was dried at ca. 443 K under dynamic vacuum for 1.5 h. The support was impregnated at room temperature under static vacuum to 95% of the total pore volume with an acidified aqueous solution containing 1.8 M copper nitrate (Acros Organics, 99%) and 0-1.8 M zinc nitrate (Sigma-Aldrich, ≥99%). Subsequently, the impregnated support was dried overnight at room temperature under dynamic vacuum and further reduced at 503 K (ramp 2 K min^{-1}) in a 100 mL min^{-1} flow of 20 vol % H_2/N_2 for 2.5 h. After cooling to room temperature, the sample was exposed to a flow of 100 mL min^{-1} flow of 5 vol % O_2/N_2 for 1 h, heated to 473 K with a ramp of 1 K min^{-1} and oxidized at 473 K in 15 vol % O_2/N_2 for 1 h.[16]

- **FollowOtherProcedure**
- DrySolid for 1.5 h at ca. 443 K under vacuum
- **ThermalTreatment reduction under static acid (1.8 M) at room temperature under (1.6 M) zinc nitrate at room temperature**
- DrySolid for overnight at room temperature under vacuum
- ThermalTreatment heat under H_2/N_2 (100 mL min^{-1})
- **NoAction**

Supported In_2O_3-based catalyst for methanol synthesis

Supported catalysts with 5 wt% In_2O_3 were attained by wet impregnation (WI) using self-prepared tetragonal zirconia (*t*-ZrO_2) and commercial monoclinic zirconia (Saint-Gobain NorPro, 95 %) and alumina (Sigma Aldrich, 99 %). To produce *t*-ZrO_2, 20 g of a $ZrO(NO_3)_2$ solution were diluted with deionized water (100 cm^3). Ethylenediamine was added dropwise (*ca*. 3 cm^3 min^{-1}) to this solution until reaching pH 10 and the resulting slurry was stirred at 353 K for 3 h. The precipitate was recovered by high-pressure filtration, washed three times with deionized water (250 cm^3 each time), dried in a vacuum oven (2 kPa, 323 K, 12 h) and calcined at 973 K (3 K min^{-1}) for 3 h in static air. WI encompassed suspending 2 g of carrier in a mixture of deionized water (54 cm^3) and ethanol (70 cm^3). The resulting slurry was magnetically stirred at 500 rpm for 12 h at room temperature. Thereafter, the solvent was removed using a rotary evaporator (Büchi Rotavap R-114) at 323 K, keeping the slurry constantly at boiling point by lowering the pressure from 180 to 40 mbar. The solid was then dried in a vacuum oven (2 kPa, 323 K, 12 h) and calcined at 773 K at 2 K min^{-1} for 3 h in static air.[17]

- **NoAction**
- **Yield t-ZrO_2**
- Add $ZrO(NO_3)_2$ solution (20 g)
- Add deionized water (100 cm^3)
- Add Ethylenediamine dropwise
- Stir for 3 h at 353 K
- Filter keep precipitate
- Wash with deionized water (250 cm^3) 3 x
- DrySolid for 12 h at **ramp**
- ThermalTreatment calcination under air for 3 h at 973 K
- MakeSolution with carrier (2 g) and deionized water (54 cm^3) and ethanol (70 cm^3)
- Stir for 12 h at room temperature

- Concentrate
- DrySolid for 12 h at 773 K under vacuum
- ThermalTreatment calcination under air for 3 h at 773 K ramp 2 K min^{-1}

ZnO/Al_2O_3 catalyst for methanol synthesis

The traditional ZnO/Al_2O_3 composite catalyst was prepared by Na_2CO_3 coprecipitation. Typically, the mixed Zn/Al solution (atomic ratios of Zn^{2+}: Al^{3+} = 0.5) and Na_2CO_3 solution (0.4 mol L^{-1}) were added dropwise to 200 mL of deionized water under vigorous stirring. The pH was kept at a constant value of 9.0 ± 0.2 during precipitation. Then, the suspension was stirred for 1 h at 70°C and aged for 2 h. The white powder was isolated by filtration, washing, and drying at 80°C overnight. Afterward, the filter cakes were calcined in air at 500°C for 4 h (heating rate 5°C min^{-1}). Following the ZnO/Al_2O_3 composite oxide was obtained, denoted as ZA-C.[18]

- **NoAction**
- MakeSolution with Zn/Al solution and **Zn^{2+} = 0.5)** and Na_2CO_3 solution (0.4 mol L^{-1}) and deionized water (200 mL)
- **Wait for unknown**
- Stir for 1 h at 70°C
- Wait for 2 h
- Filter keep precipitate
- DrySolid for overnight at 80°C
- ThermalTreatment calcination under air for 4 h at 500°C ramp 5°C min^{-1}
- **FollowOtherProcedure**

Supported Au catalyst for acetylene hydrochlorination

In this study, a loading of 1 wt% Au was used despite the commercial catalyst operating using 0.1 wt% Au. A higher loading was used in the present study to aid the characterization of fresh and used materials. Chloroauric acid (20 mg) was dissolved in aqua regia (3 HCl:1 HNO_3) (2.7 mL) and allowed to stir for 10 min. The metal precursor solution was added dropwise under stirring to ground, activated, dry carbon (0.99 g). This solution was left to stir for 1 h then dried under nitrogen at 140°C for 16 h.[19]

- **NoAction**
- NoAction
- Add Chloroauric acid (20 mg)
- Add (3 HCl:1 HNO_3) (2.7 mL)
- Stir for 10 min
- Add dry carbon (0.99 g)
- Stir for 1 h
- DrySolid for 16 h at 140°C under nitrogen

Non-metallic mercury-free catalyst for acetylene hydrochlorination

[DBU][Cl]/AC catalysts were prepared through a wetness impregnation method. Firstly, activated carbon was pretreated by HNO_3 aqueous solution (5 wt%) at 25°C to remove impurity metallic elements. Then, AC was washed to pH = 7 with deionized water, dried and reserved. Next, 1.00 g DBU and 0.65 g hydrochloric acid (5 wt%) were successively dispersed in 6.35 g anhydrous ethanol, and then the mixture was stirred for 10 min to obtain [DBU][Cl] solution. Furthermore, 5.00 g pretreated AC was added into [DBU][Cl] solution under ultrasound and the solid phase was incubated at room temperature for 8-12 h. Finally, the sample was dried at 100°C for 12 h to obtain the catalyst, named 20%[DBU][Cl]/AC.[20]

- **NoAction**
- Add **HNO_3** aqueous solution at 25°C
- Wash with **water**
- DrySolid
- Wait for unknown
- MakeSolution with DBU (1.00 g) and hydrochloric acid (0.65 g) and anhydrous ethanol (6.35 g)
- Stir for 10 min
- Yield **[rac][Cl]** solution
- Add pretreated AC (5.00 g)
- Stir for 8-12 h at room temperature
- DrySolid for 12 h at 100°C
- Yield 20%**[4']**[Cl]/AC

Supported Ru-based catalyst for acetylene hydrochlorination

Ru-MC-xN were prepared through a facile hydrothermal method. Specifically, 1 *g* MC-xN powder was sonicated in 50 mL deionized water for 10 min. Then, $RuCl_3$ of 1 wt% (in deionized water) was added drop-by-drop into the above suspension. After sonicated for 24 h, additional 10 mL 0.3 mol L^{-1} urea solution was added. Finally, the suspension was transferred to a Teflon- lined autoclave and kept at 120°C for 12 h. After cooling down naturally, the product was filtered, washed with deionized water, and centrifuged until the pH reached 7. The composites were dried at 80°C for 24 h and denoted as Ru-MC-xN.[21]

- **NoAction**
- MakeSolution with MC-*x*N powder (1 g) and deionized water (50 mL)
- Sonicate for 10 min
- Add $RuCl_3$ of 1 wt%
- Sonicate for 24 h
- Add urea solution (10 mL, 0.3 mol L^{-1})
- Transfer to Teflon-lined stainless steel autoclave
- Wait for 12 h at 120°C
- **SetTemperature** -25°C
- Filter keep precipitate
- Wash with deionized water
- **Wait** for unknown 0
- DrySolid for 24 h at 80°C
- Yield Ru-MC-*x*N

Supported Au catalyst for acetylene hydrochlorination

Au-x/AC catalysts with 1 wt% Au content were prepared by the impregnation method. For the synthesis of Au-water/AC catalyst, firstly, 1 g of $HAuCl_4 \cdot 4H_2O$ was dissolved in 50 mL water in a 50-mL volumetric flask to prepare the Au solution, in which the concentration of Au is 0.00956 g mL^{-1}. Secondly, 6.3 mL of the Au solution was transferred to a 50-mL empty beaker and 13.7 mL of water was added. Finally, 6 g of bare AC was weighed and added to the beaker under constant magnetic stirring for 24 h, followed by desiccation at 105°C under atmospheric pressure for 12 h. The obtained sample was labelled as Au-water/AC catalyst. Similarly, the other Au-x/AC catalysts can be obtained by replacing the water solvent by x during the preparation process, where x represents aqua regia, methanol, ethanol, n-propanol, isopropanol or isobutanol.[22]

- **NoAction**
- SynthesisProduct Au-water/AC catalyst
- MakeSolution with $HAuCl_4 \cdot 4H_2O$ (1g) and water (50mL)
- **Transfer to 50-mL stainless yellow**
- MakeSolution with Au solution (6.3mL) and water (13.7mL)
- **Add** Au solution (6.3mL)
- Stir for 24h at 105°C
- **Stir for 12h at 105°C**
- FollowOtherProcedure

Cu-based catalyst for acetylene hydrochlorination

An incipient impregnation method was used to prepare catalyst, and the mass percentage of Cu and TPPO was calculated based on AC. First, soak the AC in a 1 M hydrochloric acid solution in a 60°C water bath for 6 h. Then, collect the AC by filtration, wash it with deionized water to become neutrality, and dry it in an oven at 150°C. 0.5 g of ligand TPPO is added to 9 mL of ethanol. When TPPO is completely dissolved, 2.012 g of $CuCl_2 \cdot 2H_2O$ is added to the solution. Stir for half an hour to make it react completely with the ligand, and then slowly add 5 g of dried AC to the solution. Seal with plastic wrap and place in a 60°C water bath. After 6 h, remove the plastic wrap and continue heating for another 6 h. Transfer the sample to a 120°C oven and leave it to dry overnight. The resulting catalyst sample is labelled 15%Cu10%TPPO/AC. TPPO is replaced with other ligands to prepare different ligand catalysts based on the aforementioned procedure, and catalysts with different ligand contents are prepared by varying the TPPO quantity.[23]

- **NoAction**
- MakeSolution with 1 M hydrochloric acid solution and water
- Wait for 6 h at 60°C
- Filter keep precipitate
- Wash with deionized water
- DrySolid at 150°C
- Add ligand TPPO (0.5 g)
- Add ethanol (9 mL)
- **Add TPPO**
- Add $CuCl_2 \cdot 2H_2O$ (2.012 g)
- Stir for half an hour
- **SetTemperatureroom temperature**
- Add dry AC (5 g)
- **Add plastic wrap**

- **Add 60 water at 60**
- Wait for 6 h
- **SetTemperatureroom temperature**
- **Stir for 6 h**
- SynthesisProduct is labelled 15%Cu10%TPPO/AC
- FollowOtherProcedure

Carbon supported $AuCl_3$ catalyst for acetylene hydrochlorination

To synthesize the C-5 materials, we added 13.72 g of 4 nm colloidal silica solution to sucrose (6.25 g), sulfuric acid (0.71 g), H_2O (50 mL), and boric acid solutions (5.65 g). The mixtures were dried at 100°C for 6 h and at 160°C for another 6 h in an oven. The brown powder was carbonized at 900°C with N_2 flow and a heating rate of 5°C min^{-1} for 3 h. The carbonized powder was then etched with hydrofluoric acid solution to remove silica and boron species. The C-20/30/40 materials were synthesized by the same method by using silica with larger colloidal particle sizes as the template and by increasing the amount of boric acid. The $AuCl_3$/C-*x* catalysts were prepared through an incipient wetness impregnation technique using aqua regia as the solvent, as described in the literature. A solution of $HAuCl_4$·4 H_2O (Strem, 31.98 mg) in aqua regia (3.2 mL, 0.5 wt% Au) was added dropwise to the C-*x* materials (3.0 g) and then stirred at room temperature for 1 h. The $AuCl_3$/C-*x* catalysts were soaked for 24 h and dried at 140°C for 14 h.[24]

- Add 4 nm colloidal silica solution (13.72 g)
- Add sucrose (6.25 g)
- Add sulfuric acid (0.71 g)
- Add H_2O (50 mL)
- Add boric acid solutions (5.65 g)
- DrySolid for 6 h at 100°C
- Stir for 6 h at 160°C
- ThermalTreatment carbonization under N_2 for 3 h at 900°C ramp 5°C min^{-1}
- **Wash with hydrofluoric acid solution**
- **Wash with silica**
- **FollowOtherProcedure**
- **InvalidAction**
- **ThermalTreatment calcination under None for 24 h**
- DrySolid for 14 h at 140°C

Bimetallic Au-Sr catalyst for acetylene hydrochlorination

A $SrCl_2$ aqueous solution was added dropwise to the pretreated AC support under agitated stirring, and then the mixture was dipped for 10 h at room temperature, followed by evaporation at 60°C for 8 h and the quantitative addition of a solution of $HAuCl_4·4H_2O$ in *aqua regia* under agitated stirring. The mixture was dipped, evaporated, and then dried at 150°C for 14 h and used as a catalyst. The obtained bimetallic catalysts were denoted as Au1Sr(ii)*x*/AC (*x* = 0.5, 1, and 3) with a Au/Sr(ii) molar ratio of 1 : 0.5, 1 : 1, and 1 : 3, respectively.[25]

- Add $SrCl_2$ aqueous solution dropwise
- Stir for 10 h at room temperature
- Concentrate
- **Stir for 8 h at 60°C**
- Add $HAuCl_4·4H_2O$
- Concentrate
- DrySolid for 14 h at 150°C
- Yield Au1Sr(ii)x/AC

Supplementary Note 7. Model limitations

In our evaluation of the ACE transformer, we identified two prominent limitations, which are not straightforward to account for with language models. The first is the model's inability to extract grouped information. A common practice in writing synthetic protocols is to group repetitive information in a single sentence, for example, if samples are prepared with different metal contents or if they are activated at different temperatures, all the details may be collected in parenthesis as illustrated in the examples below. However, the model typically only captures one of the given values or assigns an arbitrary value.

Grouped information

0.837 g $Zn(NO_3)_2{\cdot}6H_2O$ and a certain amount of $NiCl_2{\cdot}6H_2O$ (0.1 mmol, 0.3 mmol and 0.5 mmol) were dissolved in 35 mL methanol first.

- MakeSolution with $Zn(NO_3)_2{\cdot}6H_2O$ (0.837g) and $NiCl_2{\cdot}6H_2O$ **(0.1mmol, 0.3mmol) and 0.5mmol)** and methanol (35mL)

Grouped information

The dried solid was pyrolyzed at a certain temperature (700, 800, 900, and 1000°C) for 2 h at a heating rate of 5 °C min^{-1} under flow of Ar.

- ThermalTreatment pyrolysis under Ar for 2h at **1000°C** ramp 5°C min^{-1}

The second limitation we observed is the model's inability to capture less common synthesis protocols, for example, gas-phase or electrochemical (see examples below). Based on the model prediction, it is evident that important procedures like plasma treatment and configuration of the plasma chamber for gas-phase; and details including type of electrodes, calibrations and cycles for electro are not well captured by action terms of the model and results in a NoAction as the output. The poor generalization is primarily due to the absence of these steps as action terms due to the scarcity of these methods in the training set of paragraphs for annotation. This is an important limitation to keep in mind when using the model for prediction or extraction in these areas but the model's predictive capabilities could be improved by supplementing the action terms.

Here we raise the disclaimer that the current work is only a first step towards automated extraction of action sequences for SAC synthesis. By training on 127 paragraphs primarily pertaining to solution-phase and high-temperature synthesis routes, our model is able to extract relevant information to an acceptable degree. It is worth a mention that for the above listed limitations, the former is a direct resultant of heterogeneity and lack of standardization in writing synthesis protocols, while the latter stems from scanty data for the model to train on gas-phase or electrochemical procedures. As such, we claim that with increase in training data and/or curation of action verbs specific to the abovementioned mentioned routes, the accuracy of the model should scale proportionately.

Gas-phase synthesis

The synthesis of Pt SA/C-Air catalysts involves a two-step process. 100 mg of acetylene black and 0.25 mL of 0.01 M $H_2PtCl_6 \cdot xH_2O$ solution were dispersed in 5 mL of ethanol for 2 h to obtain a homogenous black suspension. Then the slurries were putting in an oven to dry at 60°C for 12 h. After that, a black solid product was ground into a fine powder. The final product can be obtained via the plasma treatment under an air atmosphere with a flow rate of 20 mL/min. The power and vacuum of plasma devices were 80 W and 10 Pa. (Note: the plasma technique in this work is radio frequency (RF) plasma, which is powered by a 13.56 MHz power source. Before the start-up of the plasma, the chamber is vacuumized lower than 0.1 Pa. After that, the desired gas with a certain flow rate is injected continuously. In this case, the vacuum degree will decrease to around 10 Pa when the gas rate is 20 mL/min. The higher flow will lead to a lower vacuum degree. Adjust the power of the plasma emitter when the flow is steady.[26]

- NoAction
- MakeSolution with acetylene black (100 mg) and $H_2PtCl_6 \cdot xH_2O$ solution (0.25 mL) and ethanol (5 mL)
- Yield black suspension
- ThermalTreatment heat under None for 12 h at 60°C
- InvalidAction
- ThermalTreatment reduction under air for unknown
- **NoAction**
- **NoAction**
- **NoAction**
- **NoAction**
- **PH with plasma emitter**

Electrochemical synthesis

To synthesize Pd1-N8/CNT catalysts, PdO/CNT sheet was used as the working electrode. CV was carried out using computer-controlled CH Instruments 832C in a three-electrode setup. The PdO/CNT sheet was dipped in 40 mL 0.5-2 M NaN_3 (Aldrich) dissolved in a buffer solution (pH 4.0), which was used as the electrolyte. Pt and Ag/AgCl were used as the counter electrode and standard reference electrode, respectively. The CV scan rate was set to be 1 mV/s and the potential range was set to be between + 0.8 V and −0.8 V. The sample was scanned for 12 CV cycles and then dried overnight in the air. CV was also carried out on PdO/CNT sheet without NaN_3 in the solution to clarify the oxidation peak from N_3.[27]

- NoAction
- **SetTemperature 832C**
- MakeSolution with PdO/CNT sheet and NaN_3 (40 mL 0.5-2 M)
- Add solution
- **Add buffer solution**
- **NoAction**
- **NoAction**
- DrySolid for overnight under air
- NoAction

Supplementary Note 8. Model predictions with and without guidelines

In this section, we show that the predictive capabilities of the ACE model significantly improve when subjected to protocols that that are written following the guidelines suggested us. To demonstrate the same, we selected 11 synthesis paragraphs for various SACs and subjected it to the ACE model. We then modified the paragraphs based on the guidelines proposed by us and evaluated the model performance. For the examples provided below, the paragraphs are the pristine and modified paragraphs, and incorrect model predictions are highlighted in **bold**.

Synthesis of Ni/NCNTs SAC – pristine protocol

0.165 g of 2-methylimidazole was dissolved in a mixture of 12 mL DMF and 4 mL deionized water. Then, the solution was transferred into a 30 mL quartz bottle. Next, certain amounts of ZnO (0.021 g) and $Ni(NO_3)_2 \cdot 6H_2O$ (0.024 g for ZnO@ZIF-NiZn50 and 0.012 g for ZnO@ZIFNiZn100) were added to the solution, followed by sonication for 30 min. The bottle was placed in an oven for 24 h at 70°C. Finally, the obtained powder was washed with ethanol 5 times, followed by drying in a vacuum oven for 12 h at 70°C. The as-prepared core-shell ZnO@ZIF-NiZn50 was placed in an alumina crucible and transferred to a vacuum tube furnace. Before carbonization, the air in the furnace was expelled by blowing nitrogen for 1 h. Then, the furnace was heated to 200°C at a rate of 5°C min^{-1} and maintained for 2 h. The temperature was increased to 800°C, maintained for 5 h. The samples were etched with dilute HCl to remove the residual ZnO and Ni nanoparticles, followed by washing with distilled water and drying to afford Ni/NCNTs-50. Ni/NCNTs-100 was prepared using ZnO@ZIF-NiZn100 as the same procedure.[28]

- MakeSolution with DMF (12 mL) and deionized water (4 mL)
- Add 2-methylimidazole (0.165 g)
- Transfer to quartz tube (30 mL)
- **SynthesisProduct ZnO@ZIF-NiZn50**
- **MakeSolution with ZnO@ZISelectiZn100 (0.012 g) and ZnO@ZIZE raciZn (0.012 g)**
- Sonicate for 30 min
- Wait for 24 h at 70°C
- Wash with ethanol 5 x
- DrySolid for 12 h at 70°C under vacuum
- SynthesisProduct ZnO@ZIF-NiZn50
- Transfer to tube furnace
- **ThermalTeatment carbonization under nitrogen for 1 h**
- ThermalTeatment heat under None for 2 h at 200°C ramp 5°C min^{-1}

- **Stir for 5 h at 800°C**
- Add HCl
- **Filter keep precipitate**
- Wash with distilled water
- DrySolid
- Yield Ni/NCNTs-50
- FollowOtherProcedure

Synthesis of Ni/NCNTs SAC – standardized protocol

The Ni/NCNTs-50 catalyst was synthesized by solvothermal method followed by thermal treatment. 0.165 g of 2-methylimidazole was dissolved in a mixture of 12 mL DMF and 4 mL deionized water, and the solution was transferred into a 30 mL quartz bottle. Next, 0.021 g of ZnO and 0.024 g of $Ni(NO_3)_2 \cdot 6H_2O$ was added to the solution, followed by sonication for 30 min. The bottle was placed in an oven for 24 h at 70°C. Finally, the obtained powder was washed with ethanol 5 times, followed by drying in a vacuum oven for 12 h at 70°C to yield ZnO@ZIF-NiZn50. The as-prepared core-shell was placed in an alumina crucible and transferred to a vacuum tube furnace and heated to 200°C at a rate of 5°C min^{-1} and maintained for 2 h. The samples were etched with dilute HCl to remove the residual ZnO and Ni nanoparticles, followed by washing with distilled water and drying to synthesize Ni/NCNTs-50. Ni/NCNTs-100 was prepared using ZnO@ZIF-NiZn100 as the same procedure as listed in Table A.

- **NoAction**
- MakeSolution with DMF (12 mL) and water (4 mL)
- Add 2-methylimidazole (0.165 g)
- Transfer to 30 mL quartz tube
- Add ZnO (0.021 g)
- Add $Ni(NO_3)_2 \cdot 6H_2O$ (0.024 g)
- Sonicate for 30 min
- Wait for 24 h at 70°C
- Wash with ethanol 5 x
- DrySolid for 12 h at 70°C under vacuum
- Yield ZnO@ZIF-NiZn50
- **Transfer to vacuum**
- ThermalTeatment reduction under vacuum for 2 h at 200°C ramp 5°C min^{-1}
- Add HCl

- **Filter keep precipitate**
- Wash with distilled water
- DrySolid
- SynthesisProduct Ni/NCNTs-50
- FollowOtherProcedure

Synthesis of Au/CeO_2 SAC – pristine protocol

CeO_2 supported Au single-atom catalyst (SAC) was prepared by a facile adsorption method. Typically, 1 g homemade CeO_2 powder was dispersed in deionized water with rigorous stirring. Appropriate amount of $HAuCl_4$ solutions were added dropwise into the CeO_2 solution under stirring. After continuing stirring for 2 h and followed by aging for 2 h, the solution was filtered and washed with deionized water for several times, and then dried at 60°C for 5 hours without any heat treatment hereafter. The synthesized catalyst was denoted as 0.04 wt% Au/CeO_2. For comparison, an Au/CeO_2 nanoparticle (NP) catalyst consists of mainly Au NPs with mean size of 4.5 nm was purchased from Haruta Gold Inc. and denoted as Au/CeO_2 -RRCe. CeO_2 supported Pt SAC with Pt loading of 0.05 wt% was prepared by the same method to that for the Au/CeO_2 preparation except that $H_2PtCl_6 \cdot 6H_2O$ was used to substitute the $HAuCl_4 \cdot 4H_2O$, and the catalyst is denoted as Pt/CeO_2.[29]

- **NoAction**
- MakeSolution with CeO_2 (1 g) and deionized water
- Add $HAuCl_4$ solutions
- Stir for 2 h
- Wait for 2 h
- Filter keep precipitate
- Wash with deionized water 5 x
- DrySolid for 5 hours at 60°C
- SynthesisProduct 0.04 wt% Au/CeO_2
- NoAction
- FollowOtherProcedure

Synthesis of Au/CeO_2 SAC – standardized protocol

The Au/CeO_2 catalyst was synthesized by facile adsorption method Typically, 1 g homemade CeO_2 powder was dispersed in deionized water with rigorous stirring. 10 mL of $HAuCl_4$ solution were added dropwise into the CeO_2 solution under stirring. After continuing stirring for 2 h, followed by aging for 2 h, the solution was filtered, washed with deionized water for several times, and then dried at 60°C for 5 hours without any heat treatment hereafter. The synthesized catalyst was denoted as 0.04 wt% Au/CeO_2. For comparison, an Au/CeO_2 nanoparticle (NP) catalyst was purchased from Haruta Gold Inc. and denoted as Au/CeO_2 -RRCe. CeO_2 supported Pt SAC was prepared using similar procedure as listed in Table A.

- **NoAction**
- Add $HAuCl_4$ solution (10 mL)
- Stir for 2 h
- Wait for 2 h
- Filter keep precipitate
- Wash with deionized water 5 x
- DrySolid for 5 hours at 60°C
- SynthesisProduct 0.04 wt% Au/ CeO_2
- NoAction
- FollowOtherProcedure

Synthesis of Pt/C_3N_4 SAC – pristine protocol

Dicyandiamide (DCD, Sigma-Aldrich, 99%,1.2 g) was dissolved in methanol (MERCK,50 mL) and carbon black (Ketjenblack EC-600JD, 0.9g) was added to this solution. The mixture was bath-sonicated for 10 min and heated in an oil bath overnight with stirring at 80°C. The resulting powder was heat-treated at 550°C for 4 h under Ar atmosphere to obtain $C@C_3N_4$ support. Pt with the content of 1 wt%, 2 wt%, 4 wt% and 8 wt% was deposited on $C@C_3N_4$ support by wetness impregnation method. 200 mg of $C@C_3N_4$ was dispersed in 5 mL of anhydrous ethanol (MERCK) and bath-sonicated for 10 min. Pt precursor solution was prepared by dissolving an appropriate amount of chloroplatinic acid hexahydrate ($H_2PtCl_6 \cdot 6H_2O$, Sigma Aldrich) in 1 mL of anhydrous ethanol. The Pt precursor solution was added to the $C@C_3N_4$ solution and stirred until the solution was completely dried at 60°C. The resulting powder was further dried in a vacuum oven at 50°C overnight. Then, the obtained black powder was grinded softly and treated at 200°C under N_2 atmosphere.[30]

- **Add Dicyandiamide**
- Add methanol (MERCK,50 mL)
- Add carbon black (Ketjenblack EC-600JD) (0.9g)
- Sonicate for 10 min
- ThermalTeatment heat under None for overnight at 80°C
- ThermalTeatment reduction under Ar for 4 h at 550°C
- Yield $C@C_3N_4$ support
- Impregnate Pt on C@ C_3N_4 loading 1 wt%, 2 wt%, 4 wt% and 8 wt%
- MakeSolution with C@ C_3N_4 (200 mg) and anhydrous ethanol (5 mL)
- Sonicate for 10 min
- MakeSolution with chloroplatinic acid hexahydrate **($H_2PtCl_6 \cdot 6H_2O$)** and anhydrous ethanol (1 mL)
- Add Pt precursor
- Add C@ C_3N_4 solution

- DrySolution
- DrySolid for overnight at 50°C under vacuum
- ThermalTeatment reduction under N_2 at 200°C

Synthesis of Pt/C_3N_4 SAC – standardized protocol

The Pt/C_3N_4. catalyst was synthesized by wetness impregnation followed by thermal treatment. Dicyandiamide (99%) and H2PtCl6·6H2O were purchased from Sigma-Aldrich, methanol and ethanol from Merck and carbon black (EC-600JD) from Ketjenblack. Dicyandiamide (1.2 g) was dissolved in methanol (50 mL) and carbon black (0.9g) was added to this solution. The mixture was bath-sonicated for 10 min and heated in an oil bath overnight with stirring at 80°C. The resulting powder was heat-treated at 550°C for 4 h under Ar atmosphere to obtain $C@C_3N_4$. Pt with the content of 1 wt%, 2 wt%, 4 wt% and 8 wt% was deposited on $C@C_3N_4$ support by wetness impregnation method. 200 mg of $C@C_3N_4$ was dispersed in 5 mL of anhydrous ethanol and bath-sonicated for 10 min. Pt precursor solution was prepared by dissolving a 0.2mL of $H_2PtCl_6·6H_2O$ in 1 mL of anhydrous ethanol. The Pt precursor solution was added to the $C@C_3N_4$ solution and stirred until the solution was completely dried at 60°C. The resulting powder was dried in vacuum oven at 50°C overnight, and the obtained black powder was grinded softly and treated at 200°C under N_2 atmosphere to yield Pt/C_3N_4 catalyst.

- **NoAction**
- NoAction
- Add Dicyandiamide (1.2 g)
- Add methanol (50 mL)
- Add carbon black (0.9g)
- Sonicate for 10 min
- Stir for overnight at 80°C
- ThermalTeatment reduction under Ar for 4 h at 550°C
- Yield $C@C_3N_4$
- Impregnate Pt on $C@C_3N_4$ loading 1 wt%, 2 wt%, 4 wt% and 8 wt%
- MakeSolution with $C@C_3N_4$ (200 mg) and anhydrous ethanol (5 mL)
- Sonicate for 10 min

- MakeSolution with $H_2PtCl_6 \cdot 6H_2O$ (0.2mL) and anhydrous ethanol (1 mL)
- Add Pt precursor
- Add C@C_3N_4 solution
- **DrySolution**
- DrySolid for overnight at 50°C under vacuum
- **ThermalTeatment reduction under N_2**
- SynthesisProduct Pt/C_3N_4catalyst

Synthesis of Ni-N_2O_2/C SAC – pristine protocol

Nickel acetate tetrahydrate (14.8 mg) was added into 5 mL of ethanol and dissolved by stirring for 10 min. After the addition of Jacobsen's ligand (292.2 mg) into the above transparent solution, the reaction mixture was stirred continuously at 60°C for 4 h in an oil bath. Then, carbon black (69.6 mg) was added into the solution and the reaction took place for further 4 h with stirring continuously at 60°C in an oil bath. Upon drying at 80°C overnight to evaporate the ethanol and being ground in a mortar, the product was transferred into a ceramic crucible and heated in a tube furnace at 300°C for 2 h with a rate of 10°C min^{-1} under an argon atmosphere. Next, the product cooled to room temperature and was ground in a mortar. Thereafter, to remove any residual Jacobsen-Ni complex and Jacobsen ligand, the powder was dispersed in 30% acetic acid solution (40 mL) for 2 h. The ultimate product (named here as Ni-N_2O_2/C) was obtained after washing with water and ethanol and dried at 80°C overnight.[31]

- MakeSolution with Nickel acetate tetrahydrate (14.8 mg) and ethanol (5 mL)
- Stir for 10 min
- Add **benzotriazolacobsen's** ligand (292.2 mg)
- Stir for 4 h at 60°C
- Add carbon black (69.6 mg)
- Stir for 4 h at 60°C
- DrySolid for overnight at 80°C
- Concentrate
- Transfer to tube furnace under argon for 2 h at 300°C ramp 10°C min^{-1}
- SetTemperatureroom temperature
- **Add mortar**
- **Concentrate**
- Add 30% acetic acid solution (40 mL)
- Wait for 2 h

- Wash with water
- Wash with ethanol
- DrySolid for overnight at 80°C

Synthesis of Ni-N_2O_2/C SAC – standardized protocol

The Ni- N_2O/C catalyst was synthesized by solvent evaporation followed by thermal treatment. Nickel acetate tetrahydrate (14.8 mg) was added into 5 mL of ethanol and stirred for 10 min. The Jacobsen's ligand (292.2 mg) was added into the above solution and stirred continuously at 60°C for 4 h in an oil bath. The solution was dried at 80°C overnight to evaporate the ethanol, then ground using a mortar and transferred into a ceramic crucible. The fine powder was heated in a tube furnace at 300°C for 2 h with a rate of 10°C min^{-1} under an argon atmosphere. Next, the powder was cooled to room temperature and dispersed in 30% acetic acid solution (40 mL) for 2 h to remove any residual Jacobsen-Ni complex and Jacobsen ligand. The resulting powder was washed with water and ethanol and dried at 80°C overnight to yield Ni-N_2O_2/C catalyst.

- **NoAction**
- MakeSolution with Nickel acetate tetrahydrate (14.8 mg) and ethanol (5 mL)
- Stir for 10 min
- Add Jacobsens ligand (292.2 mg)
- Stir for 4 h at 60°C
- DrySolid for overnight at 80°C
- Concentrate
- Grind
- ThermalTeatment heat under argon for 2 h at 300°C ramp 10°C min^{-1}
- SetTemperatureroom temperature
- Add 30% acetic acid solution (40 mL) over 2 h
- **Concentrate**
- Wash with water
- Wash with ethanol
- DrySolid for overnight at 80°C
- Yield Ni-N_2O_2/C catalyst

Synthesis of Ni/NC SAC – pristine protocol

First, tripotassium citrate monohydrate (10 g) was calcined at 800°C under an Ar atmosphere for 1 h, with a heating rate of 10°C min^{-1}. The obtained product was centrifuged with 1 M H_2SO_4 solution several times to remove impurities and dried at 80°C overnight. Then, 12.07 g of α-d-glucose, 0.6 g of carbon, and 0.44 g of $Ni(NO_3)_2{\cdot}6H_2O$ were dissolved in deionized water (50 mL) and dispersed homogeneously by an ultrasonic for 1 h. Afterwards, the above compound was dried at 80°C for 12 h in an oven after centrifugation at 8000 rpm three times. The obtained product was well-mixed with melamine (mixture/melamine = 1:5). The obtained mixtures were carbonized at various temperatures (i.e., 800, 900, 1000, and 1100°C) under an Ar atmosphere for 2 h. The resulted materials were denoted as Ni@NxCy- 800, Ni@NxCy- 900, Ni@NxCy-1000, and Ni@NxCy-1100 catalysts, respectively. Ni@C-800 was prepared in the same way as the Ni@NxCy-800 sample, but without using melamine.[32]

- ThermalTeatment calcination under Ar for 1 h at 800°C ramp 10°C·min^{-1}
- **Wash** with H_2SO_4 solution (1 M)
- DrySolid for overnight at 80°C
- MakeSolution with α-d-glucose (12.07 g) and carbon (0.6 g) and $Ni(NO_3)_2{\cdot}6H_2O$ (0.44 g) and deionized water (50 mL)
- Sonicate for 1 h
- DrySolid for 12 h at 80°C
- **Centrifugation**
- Add melamine
- ThermalTeatment carbonization under Ar for **2 h at ramp 1100 °C**
- SynthesisProduct Ni@NxCy-800
- **MakeSolution with Ni@NxCy-900 and Ni@NxCy-1000 and Ni@NxCy-1100 catalysts**
- FollowOtherProcedure

Synthesis of Ni/NC SAC – standardized protocol

Ni/NC catalyst was synthesized by wet impregnation followed by thermal treatment. Tripotassium citrate monohydrate (10 g) was calcined at 800°C under an Ar atmosphere for 1 h, with a heating rate of 10°C·min^{-1}. Next it was centrifuged with 1 M H_2SO_4 solution several times to remove impurities and dried at 80°C overnight. Then, 12.07 g of α-d-glucose, 0.6 g of carbon, and 0.44 g of $Ni(NO_3)_2·6H_2O$ were dissolved in deionized water (50 mL) and dispersed homogeneously by an ultrasonic for 1 h. The solution was centrifuged at 8000 rpm three times, pellets collected and dried in an oven at 80°C for 12 h. The obtained product was well-mixed with melamine (mixture/melamine = 1:5) and carbonized at 800°C under an Ar atmosphere for 2 h to yield Ni/NC catalyst. Details of similar carbonization temperatures and preparation of Ni@C are listed in listed in Table A.

- **NoAction**
- ThermalTeatment calcination under Ar for 1 h at 800°C ramp 10°C·min^{-1}
- **Wash with 1 M H_2SO_4 solution**
- DrySolid for overnight at 80°C
- MakeSolution with α-d-glucose (12.07 g) and carbon (0.6 g) and $Ni(NO_3)_2·6H_2O$ (0.44 g) and deionized water (50 mL)
- Sonicate for 1 h
- Centrifuge rpm 8000
- Filter keep precipitate
- DrySolid for 12 h at 80°C
- **Add melamine over 2 h**
- Yield Ni/NC catalyst
- NoAction

Synthesis of W/NC SAC – pristine protocol

2-Methylimidazole (2 g) (Shanghai Chemical Reagents Co. Ltd) was dissolved in 10 mL of methanol (Sinopharm Chemical Reagents Co. Ltd), then 10 mL of methanol solution with $Zn(NO_3)_2 \cdot 6H_2O$ (1.8 g) (Aladdin Company) and WCl_6 (8 mg) (Aladdin Company) was added under stirring. The white crystal of W-ZIF-8 was formed after continuous stirring at normal temperature for 20 h. After washing and isolation by centrifugation, the obtained W-ZIF-8 precursor was dried at 80°C for 5 h. For comparison, the pristine ZIF-8 without W atom was synthesized by the same procedure except for the addition of WCl_6. ZIF-8 precursors and W-ZIF-8 precursors were separately heated to 1000°C in an argon flow and kept for 120 min and then cooled with the furnace. Then, the carbonized materials were washed with 0.3 M HCl for removing the residual metal elements. After washing three times with deionized water, the obtained materials, named N-C and W-N-C, were further dried at 80°C overnight for characterization.[33]

- Add 2-Methylimidazole (2 g)
- Add methanol (10 mL)
- Add $Zn(NO_3)_2 \cdot 6H_2O$ (1.8 g)
- **Add Aladdin (8 mg)**
- Stir for 20 h at 32°C
- Centrifuge
- DrySolid for 5 h at 80°C
- FollowOtherProcedure
- ThermalTeatment heat under argon for 120 min at 1000°C
- **SetTemperature furnace**
- Wash with HCl (0.3 M)
- **Filter keep filtrate**
- Wash with deionized water 3 x
- **Wash with N-C**

- DrySolid for overnight at 80°C

Synthesis of W/NC SAC – standardized protocol

W/NC catalyst was synthesized by chemical precipitation followed by thermal treatment. 2- Methylimidazole and methanol was procured from Sinopharm Chemical Reagents Co. Ltd, while $Zn(NO_3)_2·6H_2O$ and WCl_6 were purchased from Aladdin company. 2-Methylimidazole (2 g) was dissolved in 10 mL of methanol. A solution containing $Zn(NO_3)_2·6H_2O$ (1.8 g) and WCl_6 (8 mg) in 10 mL of methanol with was added to the above solution and stirred for 20 h. The solution was centrifuged, washed with water, and dried at 80°C for 5 h to yield W-ZIF-8 precursor. The W-ZIF-8 precursors was heated to 1000°C in an argon flow and kept for 120 min and then cooled with the furnace. Finally, the carbonized materials were washed with 0.3 M HCl, then with deionized water three time, and dried at 80°C overnight to yield W/NC catalyst. NC was prepared in a similar procedure and the details are listed in Table A.

NoAction

NoAction

MakeSolution with 2-Methylimidazole (2 g) and methanol (10 mL)

MakeSolution with $Zn(NO_3)_2·6H_2O$ (1.8 g) and WCl_6 (8 mg) and methanol (10 mL)

Add SLN

Stir for 20 h

Centrifuge

Wash with water

DrySolid for 5 h at 80°C

Yield N-ZIF-8 precursor

ThermalTeatment heat under argon for 120 min at 1000°C

SetTemperaturefurnace

Wash with HCl (0.3 M)

Wash with deionized water 3 x

DrySolid for overnight at 80°C

Yield W/NC catalyst

FollowOtherProcedure

Synthesis of Pd_1Zn/ZnO SAC – pristine protocol

Pd_1Zn_3/ZnO was synthesized via a modified deposition precipitation method by anchoring the Pd cations of $Pd(NO_3)_2$ on commercial ZnO (Alfa, >99%, nanoparticles with sizes of 20-30 nm). Preparation of catalyst precursors is prerequisite for synthesis of catalysts (0.01 wt% Pd/ZnO and 0.05 wt% Pd/ZnO) with isolated Pd_1Zn_3 bimetallic sites, which was done through the following steps. A suspension was obtained by mixing ZnO with 50 mL deionized water under vigorous stirring. Then, certain amount of $Pd(NO_3)_2$ solution was added through a controlled injection with a syringe pump when the ZnO suspension was being vigorously stirred. Then, pH of the suspension was adjusted carefully to 9.5 by gradually adding ammonium hydroxide solution, followed by continuously vigorous stirring overnight. Then, the solution was centrifuged and washed with deionized water several times, offering a precursor for preparation of catalyst, 0.01 wt% Pd/ZnO or 0.05 wt% Pd/ZnO.[34]

- **NoAction**
- NoAction
- Add ZnO
- Add deionized water (50 mL)
- Add $Pd(NO_3)_2$ solution
- Stir
- Ph with ammonium hydroxide solution to pH 9.5
- Stir for overnight
- Centrifuge
- Wash with water 5 x
- **Catalyst name**

Synthesis of Pd_1Zn/ZnO SAC – standardized protocol

The Pd_1Zn_3/ZnO was synthesized via modified deposition precipitation. Commercial ZnO (>99%, nanoparticles with sizes of 20-30 nm) was procured from Alfa. Mix ZnO with 50 mL deionized water under vigorous stirring to create a suspension. Then, add 5mL of $Pd(NO_3)_2$ solution through a controlled injection with a syringe pump while the ZnO suspension is vigorously stirred. Then, pH of the suspension was adjusted carefully to 9.5 by gradually adding ammonium hydroxide solution, followed by continuously vigorous stirring overnight. Then, the solution was centrifuged and washed with deionized water several times to obtain Pd_1Zn_3/ZnO.

- **NoAction**
- **Yield ZnO (>99%, nanoparticles of 20-30 nm)**
- MakeSolution with ZnO and deionized water (50 mL)
- Stir
- Add $Pd(NO_3)_2$ solution (5mL)
- Stir
- pH with ammonium hydroxide solution to pH 9.5
- Stir for overnight
- Centrifuge
- Wash with water 5 x
- Yield Pd_1Zn_3/ZnO

Synthesis of Pt/CeO*x*-TiO_2 SAC – pristine protocol

To synthesize CeOx-TiO_2 hybrid-oxides and nPT and nPCT catalysts, titanium (IV) oxide (anatase, #45603, Alfa Aesar) powder, cerium (III) nitrate hexahydrate ($CeN_3O_9 \cdot 6H_2O$, #11330, Alfa Aesar) powder, and chloroplatinic acid (H_2PtCl_8 8 wt% in H_2O, Sigma-Aldrich) solution were used. As-received anatase- TiO_2 powders were heat-treated under air at 500°C for 4 hours before use. CeOx-TiO_2 oxide supports were synthesized by the wet impregnation method. Initially, 1 g of TiO_2 powder and varying contents of Ce precursor were dispersed in 60 ml of deionized (DI) water and stirred for 2 hours at 70°C. The initial amount of Ce was set to 1 wt% with respect to the calculated amount of Ti ions in the TiO_2 powder. The solution was dried at 110°C for 12 hours and calcined in air at 500°C for 8 hours with a heating rate of 2°C min^{-1}. To synthesize nPCT and nPT catalysts, varying amounts of H_2PtCl_8 solution was added to 60 mL of deionized water with 1 g of CeOx-TiO_2 or TiO_2 powder. The initial amount of Pt was set to 0.25, 0.5, and 1.0 wt% with respect to the total weight of nPCT or nPT catalysts. After Pt impregnation, the resulting samples were dried at 110°C for 12 hours and calcined in air at 500°C for 3 hours to remove the residual chlorine.[35]

- **InvalidAction**
- ThermalTeatment reduction under air for 4 hours at 500°C
- **NoAction**
- MakeSolution with TiO_2 powder (1 g) and water (60 ml)
- Stir for 2 hours at 70°C
- NoAction
- DrySolid for 12 hours at 110°C
- ThermalTeatment calcination under air for 8 hours at 500°C ramp 2°C min^{-1}
- **MakeSolution with nPCT and nPT catalysts**
- Add H_2PtCl_8 solution
- Add deionized water (60 mL)
- Add CeO*x*-TiO_2 or TiO_2 (1 g)

- **NoAction**
- **SynthesisProduct Pt impregnation**
- DrySolid for 12 hours at 110°C
- ThermalTeatment calcination under air for 3 hours at 500°C

Synthesis of Pt/CeO_x-TiO_2 SAC – standardized protocol

The Pt/CeOx-TiO_2 catalyst was prepared by wet-impregnation followed by thermal treatment. Titanium (IV) oxide (Anatase, #45603), and cerium (III) nitrate hexahydrate (CeN_3O_9·6H2O, #11330) were procured from Alfa Aesar while chloroplatinic acid (H_2PtCl_8) was purchased from Sigma-Aldrich. As-received anatase- TiO_2 powders were heat-treated under air at 500°C for 4 hours before use. 1 g of TiO_2 powder and varying contents of Ce precursor were dispersed in 60 ml of deionized (DI) water and stirred for 2 hours at 70°C. The initial amount of Ce was set to 1 wt% with respect to the calculated amount of Ti ions in the TiO_2 powder. The solution was dried at 110°C for 12 hours and calcined in air at 500°C for 8 hours with a heating rate of 2°C min^{-1}. The as obtained CeOx-TiO_2 was impregnated with 0.25 wt% Pt. The resulting samples were dried at 110°C for 12 hours and calcined in air at 500°C for 3 hours to remove the residual chlorine. Details of nPT and nPCT catalysts prepared using similar procedure are listed in Table A.

- **SynthesisProduct Pt/ CeO_x-TiO_2 catalyst**
- NoAction
- **ThermalTeatment reduction under air for 4 hours at 500°C ramp 0°C**
- ThermalTeatment heat under air for 4 hours at 500°C
- MakeSolution with TiO_2 powder (1 g) and water (60 ml)
- Stir for 2 hours at 70°C
- NoAction
- DrySolid for 12 hours at 110°C
- ThermalTeatment calcination under air for 8 hours at 500°C ramp 2°C min^{-1}
- Yield CeO_x-TiO_2
- **Grind**
- DrySolid for 12 hours at 110°C
- ThermalTeatment calcination under air for 3 hours at 500°C
- Yield Pt/ CeO_x-TiO_2 catalyst

- FollowOtherProcedure

Synthesis of Ru/mono-NiFe SAC – pristine protocol

The mono-NiFe was prepared via one-step coprecipitation method according to the reference with slightly modification. A 20.0 mL solution composed of 0.75 mmol $Ni(NO_3)_2 \cdot 6H_2O$ and 0.25 mmol $Fe(NO_3)_3 \cdot 9H_2O$ was added drop by drop to a solution of 20.0 mL $NaNO_3$ (0.010 M) containing 23 vol% formamide. Simultaneously, 0.25 M NaOH was added dropwise to maintain the system at a pH value of ca. 10 under magnetic stirring at 80°C. The reaction was completed within 10 min. The precipitates were collected by centrifugation, washed with water and ethanol for more than 3 times. The prepared catalysts were denoted as Ru_1/mono-NiFe-x (x = 0.3, 1.6, 3.8, 7.0) corresponding to the $RuCl_3 \cdot 3H_2O$ amount of 3.75 μmol, 22.5 μmol, 62.5 μmol and 125 μmol, respectively.[36]

- FollowOtherProcedure
- **NoAction**
- **NoAction**
- Wait for 10 min
- Centrifuge
- Wash with water
- Wash with ethanol 3 x
- Yield Ru1/mono-NiFe-x (x = 0.3) **(3.75 μmol, 22.5 μmol, 62.5 μmol)**

Synthesis of Ru/mono-NiFe SAC – standardized protocol

The 0.3% Ru/mono-NiFe catalyst were prepared by modified wet-impregnation method. 0.75 mmol $Ni(NO_3)_2{\cdot}6H_2O$ and 0.25 mmol $Fe(NO_3)_3{\cdot}9H_2O$ were mixed to form a 20 mL solution. This solution was added dropwise to 20.0 mL solution of NaNO3 (0.010 M) containing 23 vol% formamide. 0.25 M NaOH was added dropwise to maintain the system at a pH value of 10 under magnetic stirring at 80°C. The reaction was completed within 10 min. The precipitates were collected by centrifugation, washed with water and ethanol for more than 3 times to. The as obtained mono-NiFe support was impregnated with 3.75 µmol of $RuCl_3{\cdot}3H_2O$ to yield 0.3% Ru/mono-NiFe. Details of other catalyst synthesized in similar procedure are listed in Table A.

- **FollowOtherProcedure**
- **MakeSolution with $Ni(NO_3)_2{\cdot}6H_2O$ (0.75 mmol) and $Fe(NO_3)_3{\cdot}9H_2O$ (0.25 mmol)**
- MakeSolution with $NaNO_3$ (0.010 M) and 23 vol% formamide (20.0 mL)
- Add SLN
- pH with NaOH (0.25 M) to pH 10 at 80°C
- Wait for 10 min
- Centrifuge
- Wash with water
- Wash with ethanol 3 x
- Yield Ru/mono-NiFe
- NoAction

Synthesis of Cu/H-ZSM-5 SAC – pristine protocol

The same synthesis strategy was used to prepare the H-ZSM-5 supported single Cu atom catalysts. Typically, H-ZSM-5 (SiO_2/Al_2O_3 = 25, the BET surface area of ca. 310 m^2g^{-1}; Tianjin Yuanli Chemical Co.) was dispersed in deionized water. The pH of Cu precursor ($CuCl_2 \cdot 2H_2O$; Sinopharm Chemical Reagent Co., China) was finely tuned from 3.5 to 4.0. The diluted Cu precursors were pumped into H-ZSM-5 suspension at a speed of ~ 0.5 mL min^{-1} under stirring (the concentration of Cu species was controlled to be less than 0.8 mmol L^{-1}). After aging for 2 h at room temperature, the sample was centrifuged and washed, then dried at 60°C in an oven overnight and calcined at 300°C for 4 h in static air. To exclude the possible influence of chloride ions, the catalysts were thoroughly washed by deionized water until no Cl- ions could be detected by saturated $AgNO_3$ solution. The Cu loading was increased to 1.50 wt% (denoted as 1.5Cu/ZSM-5) following the same procedures by increasing the concentration of Cu precursors.[37]

- **NoAction**
- **NoAction**
- **InvalidAction Conversion failed**
- **NoAction**
- **Add H-ZSM-5 suspension**
- **Stir**
- Centrifuge
- Wash with unknown
- DrySolid for overnight at 60°C
- ThermalTeatment calcination under air for 4 h at 300°C
- **InvalidAction**
- FollowOtherProcedure

Synthesis of Cu/H-ZSM-5 SAC – standardized protocol

The 1% Cu/H-ZSM-5 catalyst was prepared by impregnation and thermal treatment. H-ZSM-5 (SiO_2/Al_2O_3 = 25, the BET surface area of ca. 310 m^2g^{-1}) was procured from Tianjin Yuanli Chemical Co while $CuCl_2 \cdot 2H_2O$ was obtained from Sinopharm Chemical Reagent Co. Typically, H-ZSM-5 (2 mg) was dispersed in 5 mL deionized water and 0.4 mL of $CuCl_2 \cdot 2H_2O$ was added to it.The pH $CuCl_2 \cdot 2H_2O$ was adjusted to be between 3.5 to 4.0. After aging for 2 h at room temperature, the sample was centrifuged, washed, and dried at 60°C in an oven overnight. The sample was calcined at 300°C for 4 h in static air and finally washed by deionized water to yield 1% Cu/H-ZSM-5. Details of other catalysts prepared in similar manner are given in Table A.

- **NoAction**
- NoAction
- MakeSolution with H-ZSM-5 (2 mg) and deionized water (5 mL) and $CuCl_2 \cdot 2H_2O$ (0.4 mL)
- **pH with $CuCl_2 \cdot 2H_2O$ (0.4 mL) to pH neutral**
- Centrifuge
- Wash
- DrySolid for overnight at 60°C
- ThermalTeatment calcination under air for 4 h at 300°C
- Wash with deionized water
- Yield Cu/H-ZSM-5
- NoAction

Synthesis of Pt/$Ce_{0.7}Zr_{0.3}O_2$ SAC – pristine protocol

CeO_2 and $Ce_{0.7}Zr_{0.3}O_2$ catalyst supports were prepared by the sol-gel method. $Ce(NO_3)_3.6H_2O$ and $ZrO(NO_3)_2{\cdot}H_2O$ (used for $Ce_{0.7}Zr_{0.3}O_2$) were dissolved (molar ratio Ce:Zr = 7:3) in deionized water and citric acid was added dropwise and the resulting solution was stirred at 65°C for 2 h. The solution was then kept at 80°C in water bath until a gel was formed. After dried (overnight), the gel was calcined at 500°C for 3 h in a muffle furnace. Pt/Al_2O_3, Pt/CeO_2 and Pt/ $Ce_{0.7}Zr_{0.3}O_2$ catalysts were prepared by the wet-impregnation method. The supports of Al_2O_3, CeO_2 and $Ce_{0.7}Zr_{0.3}O_2$ were immersed into an appropriate volume of $H_2PtCl_6.6H_2O$ solution so as to provide 1 wt% Pt nominal loading and stirred at 40°C for 2 h. After evaporation at 80°C, the samples were dried overnight and calcined at 500°C for 3 h.[38]

- **NoAction**
- MakeSolution with $Ce(NO_3)_3.6H_2O$ and $ZrO(NO_3)_2{\cdot}H_2O$ and deionized water and citric acid
- **Add SLN dropwise**
- Stir for 2 h at 65°C
- SetTemperature80°C
- DrySolid for overnight
- ThermalTeatment calcination under None for 3 h at 500°C
- FollowOtherProcedure
- **MakeSolution with Al_2O_3, CeO_2 and $Ce_{0.7}Zr_{0.3}O_2$ and $H_2PtCl_6.6H_2O$ solution**
- Yield Pt **nominal**
- Concentrate
- **DrySolid** for overnight at 500°C
- **ThermalTeatment** calcination under None for 3 h at 500°C

Synthesis of Pt/$Ce_{0.7}Zr_{0.3}O_2$ SAC – standardized protocol

1% Pt/ $Ce_{0.7}Zr_{0.3}O_2$ catalyst was prepared by wet impregnation method. $Ce(NO_3)_3.6H_2O$ (1 mL) and $ZrO(NO_3)_2 \cdot H_2O$ (0.3 mL) was dissolved in 10 mL of deionized water. Citric acid (0.5 mL) was added dropwise and the resulting solution was stirred at 65°C for 2 h and kept at 80°C in water bath for overnight until a gel was formed. The gel was calcined at 500°C for 3 h in a muffle furnace. The as obtained $Ce_{0.7}Zr_{0.3}O_2$ support was immersed into 0.8 mL of $H_2PtCl_6.6H_2O$ solution and stirred at 40°C for 2 h. After evaporation at 80°C, the samples were dried overnight and calcined at 500°C for 3 h to yield 1% Pt/ $Ce_{0.7}Zr_{0.3}O_2$. Details of preparation of Pt/Al_2O_3, Pt/CeO_2 are listed in Table A.

- **FollowOtherProcedure**
- MakeSolution with $Ce(NO_3)_3.6H_2O$ (1 mL) and $ZrO(NO_3)_2 \cdot H_2O$ (0.3 mL) and deionized water (10 mL)
- Add Citric acid (0.5 mL) dropwise
- Stir for 2 h at 65°C
- **Stir for overnight at 80°C**
- ThermalTeatment calcination under None for 3 h at 500°C
- Yield $Ce_{0.7}Zr_{0.3}O_2$ support
- Add $H_2PtCl_6.6H_2O$ solution (0.8 mL)
- Stir for 2 h at 40°C
- DrySolid for overnight at 80°C
- ThermalTeatment calcination under None for 3 h at 500°C
- Yield Pt/ $Ce_{0.7}Zr_{0.3}O_2$
- NoAction

References

1. Ji, S. *et al.* Chemical synthesis of single atomic site catalysts. *Chem. Rev.* **120**, 11900–11955 (2020).
2. Chen, Y. *et al.* Single-atom catalysts: synthetic strategies and electrochemical applications. *Joule* **2**, 1242–1264 (2018).
3. Kaiser, S. K., Chen, Z., Faust Akl, D., Mitchell, S. & Pérez-Ramírez, J. Single-atom catalysts across the periodic table. *Chem. Rev.* **120**, 11703–11809 (2020).
4. Vaucher, A. C. *et al.* Automated extraction of chemical synthesis actions from experimental procedures. *Nat. Commun.* **11**, 3601 (2020).
5. Klein, G., Kim, Y., Deng, Y., Senellart, J. & Rush, A. OpenNMT: open-source toolkit for neural machine translation. *Proceedings of ACL 2017, System Demonstrations* 67–72 (Association for Computational Linguistics, 2017).
6. Schwaller, P. *et al.* Mapping the space of chemical reactions using attention-based neural networks. *Nat. Mach. Intell.* **3**, 144–152 (2021).
7. Han, X. *et al.* Atomically dispersed binary Co-Ni sites in nitrogen-doped hollow carbon nanocubes for reversible oxygen reduction and evolution. *Adv. Mater.* **31**, 1905622 (2019).
8. Li, W. *et al.* The effect of chlorine modification of precipitated iron catalysts on their Fischer-Tropsch synthesis properties. *Catalysts* **12**, 812 (2022).
9. Chen, Y. *et al.* Carbon-supported Fe catalysts with well-defined active sites for highly selective alcohol production from Fischer-Tropsch synthesis. *Appl. Catal. B Environ.* **312**, 121393 (2022).
10. Gong, K. *et al.* Carbon-encapsulated metallic Co nanoparticles for Fischer-Tropsch to olefins with low CO_2 selectivity. *Appl. Catal. B Environ.* **316**, 121700 (2022).
11. Lee, S. *et al.* Selective olefin production on silica based iron catalysts in Fischer-Tropsch synthesis. *Catal. Sci. Technol.* **12**, 5814–5828 (2022).
12. An, Y. *et al.* Effects of alkaline-earth metals on CoMn-based catalysts for the Fischer-Tropsch synthesis to olefins. *Catal. Sci. Technol.* **12**, 2677–2687 (2022).

13. Yang, Y. *et al.* Effect of the Zr promoter on precipitated iron-based catalysts for high-temperature Fischer-Tropsch synthesis of light olefins. *Catal. Sci. Technol.* **12**, 4624–4636 (2022).
14. Yang, H. *et al.* Selective synthesis of olefins via CO_2 hydrogenation over transition-metal doped iron-based catalysts. *Appl. Catal. B Environ.* **321**, 122050 (2023).
15. Zhang, S. *et al.* Morphological modulation of Co_2C by surface-adsorbed species for highly effective low-temperature CO_2 reduction. *ACS Catal.* **12**, 8544–8557 (2022).
16. Dalebout, R. *et al.* Insight into the nature of the ZnOx promoter during methanol synthesis. *ACS Catal.* **12**, 6628–6639 (2022).
17. Araújo, T. P. *et al.* Impact of hybrid CO_2-CO feeds on methanol synthesis over In_2O_3-based catalysts. *Appl. Catal. B Environ.* **285**, 119878 (2021).
18. Song, L., Wang, H., Wang, S. & Qu, Z. Dual-site activation of H_2 over $Cu/ZnAl_2O_4$ boosting CO_2 hydrogenation to methanol. *Appl. Catal. B Environ.* **322**, 122137 (2023).
19. Pattisson, S. *et al.* Lowering the operating temperature of gold acetylene hydrochlorination catalysts using oxidized carbon supports. *ACS Catal.* **12**, 14086–14095 (2022).
20. Dong, X. *et al.* Activated carbon supported nitrogen-containing diheterocycle mercury-free catalyst for acetylene hydrochlorination. *Mol. Catal.* **525**, 112366 (2022).
21. Hu, J. *et al.* Enhanced catalytic performance of oxidized Ru supported on N-doped mesoporous carbon for acetylene hydrochlorination. *Appl. Catal. Gen.* **623**, 118236 (2021).
22. Zhang, C. *et al.* Hydrochlorination of acetylene catalyzed by activated carbon supported highly dispersed gold nanoparticles. *Appl. Catal. Gen.* **566**, 15–24 (2018).
23. Wang, B. *et al.* Phosphine-oxide organic ligand improved Cu-based catalyst for acetylene hydrochlorination. *Appl. Catal. Gen.* **630**, 118461 (2022).
24. Chen, K., Kang, L., Zhu, M. & Dai, B. Mesoporous carbon with controllable pore sizes as a support of the $AuCl_3$ catalyst for acetylene hydrochlorination. *Catal. Sci. Technol.* **5**, 1035–1040 (2015).

25. Li, G., Li, W. & Zhang, J. Strontium promoted activated carbon-supported gold catalysts for non-mercury catalytic acetylene hydrochlorination. *Catal. Sci. Technol.* **6**, 3230–3237 (2016).

26. Li, J. *et al.* Cold-plasma technique enabled supported Pt single atoms with tunable coordination for hydrogen evolution reaction. *Appl. Catal. B Environ.* **285**, 119861 (2021).

27. Hu, M. *et al.* N_8 stabilized single-atom Pd for highly selective hydrogenation of acetylene. *J. Catal.* **395**, 46–53 (2021).

28. Hou, Y., Liang, Y.-L., Shi, P.-C., Huang, Y.-B. & Cao, R. Atomically dispersed Ni species on N-doped carbon nanotubes for electroreduction of CO_2 with nearly 100% CO selectivity. *Appl. Catal. B Environ.* **271**, 118929 (2020).

29. Li, T. *et al.* Maximizing the number of interfacial sites in single-atom catalysts for the highly selective, solvent-free oxidation of primary alcohols. *Angew. Chem. Int. Ed.* **57**, 7795–7799 (2018).

30. Shin, S. *et al.* Seemingly negligible amounts of platinum nanoparticles mislead electrochemical oxygen reduction reaction pathway on platinum single-atom catalysts. *ChemElectroChem* **7**, 3716–3719 (2020).

31. Wang, Y. *et al.* High-efficiency oxygen reduction to hydrogen peroxide catalyzed by nickel single-atom catalysts with tetradentate N_2O_2 coordination in a three-phase flow cell. *Angew. Chem. Int. Ed.* **59**, 13057–13062 (2020).

32. Yang, X. *et al.* Boosting electrochemical CO_2 reduction by controlling coordination environment in atomically dispersed Ni@NxCy catalysts. *ACS Sustain. Chem. Eng.* **9**, 6438–6445 (2021).

33. Jiang, B. *et al.* Framework-derived tungsten single-atom catalyst for oxygen reduction reaction. *Energy Fuels* **35**, 8173–8180 (2021).

34. Tang, Y. *et al.* Atomic-scale structure and catalysis on positively charged bimetallic sites for generation of H_2. *Nano Lett.* **20**, 6255–6262 (2020).

35. Yoo, M. *et al.* A tailored oxide interface creates dense Pt single-atom catalysts with high catalytic activity. *Energy Environ. Sci.* **13**, 1231–1239 (2020).

36. Wang, Z. *et al.* Single Ru atoms with precise coordination on a monolayer layered double hydroxide for efficient electrooxidation catalysis. *Chem. Sci.* **10**, 378–384 (2019).

37. Tang, X. *et al.* Direct oxidation of methane to oxygenates on supported single Cu atom catalyst. *Appl. Catal. B Environ.* **285**, 119827 (2021).

38. Wu, Q. *et al.* Insight of Pt-support interaction in S-Pt/$Ce_{0.7}Zr_{0.3}O_2$ by in situ Raman spectroscopy. *Catal. Commun.* **98**, 34–37 (2017).
